# Donor-Recipient Matching for KIR Genotypes Reduces Chronic GVHD and Missing Inhibitory KIR Ligands Protect against Relapse after Myeloablative, HLA Matched Hematopoietic Cell Transplantation

**DOI:** 10.1371/journal.pone.0158242

**Published:** 2016-06-24

**Authors:** Rehan Mujeeb Faridi, Taylor J. Kemp, Poonam Dharmani-Khan, Victor Lewis, Gaurav Tripathi, Raja Rajalingam, Andrew Daly, Noureddine Berka, Jan Storek, Faisal Masood Khan

**Affiliations:** 1 Department of Pathology and Laboratory Medicine, University of Calgary, Calgary, Alberta, Canada; 2 Section of Pediatric Oncology, Blood and Marrow Transplant, Department of Oncology and Pediatrics, Alberta Children's Hospital, University of Calgary, Calgary, Alberta, Canada; 3 Immunogenetics and Transplantation Laboratory, Department of Surgery, University of California San Francisco, San Francisco, California, United States of America; 4 Departments of Medicine and Oncology, Foothills Hospital and Tom Baker Cancer Centre, Calgary, Alberta, Canada; 5 Calgary Laboratory Services, University of Calgary, Calgary, Alberta, Canada; Beth Israel Deaconess Medical Center, Harvard Medical School, UNITED STATES

## Abstract

**Background:**

Allogeneic hematopoietic cell transplantation (HCT) can be curative for many hematologic diseases. However, complications such as graft-versus-host disease (GVHD) and relapse of primary malignancy remain significant and are the leading causes of morbidity and mortality. Effects of killer Ig-like receptors (KIR)-influenced NK cells on HCT outcomes have been extensively pursued over the last decade. However, the relevance of the reported algorithms on HLA matched myeloablative HCT with rabbit antithymocyte globulin (ATG) is used for GVHD prophylaxis remains elusive. Here we examined the role of KIR and KIR-ligands of donor-recipient pairs in modifying the outcomes of ATG conditioned HLA matched sibling and unrelated donor HCT

**Methods and Findings:**

The study cohort consisted of 281 HLA matched sibling and unrelated donor-recipient pairs of first allogeneic marrow or blood stem cell transplantation allocated into ‘discovery’ (135 pairs) and ‘validation’ (146 pairs) cohorts. High resolution HLA typing was obtained from the medical charts and KIR gene repertoires were obtained by a Luminex^®^ based SSO method. All surviving patients were followed-up for a minimum of two years. KIR and HLA class I distributions of HCT pairs were stratified as per applicable definitions and were tested for their association with cause specific outcomes [acute GVHD grade II-IV (aGVHD), chronic GVHD needing systemic therapy (cGVHD) and relapse] using a multivariate competing risks regression model as well as with survival outcomes [relapse-free survival (RFS), cGVHD & relapse free survival (cGRFS) and overall survival (OS)] by multivariate Cox proportional hazards regression model. A significant association between KIR genotype mismatching (KIR-B/x donor into KIR-AA recipient or vice versa) and cGVHD was found in both discovery (p = 0.001; SHR = 2.78; 95%CI: 1.50–5.17) and validation cohorts (p = 0.005; SHR = 2.61; 95%CI: 1.33–5.11). High incidence of cGVHD associated with KIR genotype mismatching was applicable to both sibling and unrelated donors and was specific to recipients who had one or two C1 bearing HLA-C epitopes (HLA-C1/x, p = 0.001; SHR = 2.40; 95%CI: 1.42–4.06). When compared with KIR genotype mismatched transplants, HLA-C1/x patients receiving grafts from KIR genotype matched donors had a significantly improved cGRFS (p = 0.013; HR = 1.62; 95%CI: 1.11–2.39). Although there was no effect of KIR genotype matching on survival outcomes, a significantly reduced incidence of relapse (p = 0.001; SHR = 0.22; 95%CI: 0.10–0.54) and improved relapse-free survival (p = 0.038; HR = 0.40; 95%CI: 0.17–0.95) was observed with one or more missing ligands for donor inhibitory KIR among the recipients of unrelated donor transplants.

**Conclusions:**

The present study for the first time presents the beneficial effects of KIR genotype matching in reducing cGVHD in myeloablative transplant setting using HLA matched (sibling and unrelated) donors. The findings offer a clinically applicable donor selection strategy that can help control cGVHD without affecting the risk of relapse and/or identify patients at a high risk of developing cGVHD as potential candidates for preemptive therapy. The findings also affirm the beneficial effect of one or more missing inhibitory KIR ligands in the recipient in reducing relapse and improving a relapse free survival in unrelated donor transplants.

## Introduction

Allogeneic hematopoietic cell transplantation (HCT) is a curative therapy for hematologic malignancies, particularly leukemia, as well as for life-threatening congenital and acquired disorders of hematolymphopoiesis [1]. Unfortunately, complications of HCT are substantial, particularly graft-vs-host disease (GVHD) and relapse of the underlying disease. Owing to a common immunologic mechanism, GVHD is strongly associated with graft versus leukemia effect (GVL) that prevents disease relapse [2]. Aimed at *in vivo* T-cell depletion, pre-transplant administration of polyclonal antithymocyte globulin (ATG) has been effective in reducing graft rejection and GVHD without increasing relapse [3]. Increasing relevance of ATG in pre-transplant preparative regimen is affirmed by five randomized studies and multiple nonrandomized studies showing the benefits of ATG in reducing aGVHD and cGVHD (reviewed by Storek et al) [4]. However, in spite of routinely using ATG in addition to high resolution HLA matching, ~40% adult peripheral blood stem cell recipients in our experience develop clinically significant GVHD. Majority of these patients fail to achieve a sustained complete response to immunosuppressive drugs and either die or experience poor quality of life due to chronic GVHD (cGVHD). Thus, it is important to characterize immunogenetic determinants other than HLA that can moderate GVHD causing allo-immune responses without limiting GVL.

In this context, regulation of natural killer (NK) cells through killer Ig-like receptors (KIR) has been the subject of intensive research. NK cells constitute a critical component of innate immunity, being the first in the line of defense against tumors and viral infections [5]; are able to suppress or amplify T cell alloreactivity [6]; and are among the earliest lymphocyte subsets to reconstitute and achieve functional maturity (within weeks) after HCT [7]. KIR encoded activating and inhibitory receptors regulate NK cell functions and help identify unhealthy targets from the healthy self-cells [5] by their recognition of HLA class-I antigens [8–11]. Genomic variation within the KIR gene cluster mapped at chromosome 19q13.4 has led to presence/absence of individual KIR loci resulting in diverse and distinct KIR ‘haplotypes’. These haplotypes are classified into two groups- A and B [12]. The Group A haplotype has a fixed set of nine genes with one activating gene (KIR2DS4) while the group B haplotypes are more diverse and carry additional activating genes. A KIR ‘genotype’ refers to the presence of two such haplotypes, which could either be B/x (A/B or B/B) or A/A. Ligands of KIR are contributed by four polymorphic epitopes of classical HLA class-I antigens, which are defined by amino acid substitutions in residues 76–83 of the α1 helix of the HLA class-I H chain. The C1 and C2 epitopes are carried by different HLA-C allotypes and are recognized by KIR2DL2/2DL3 and 2DL1/2DS1 respectively. The inhibitory KIR3DL1 recognizes the Bw4 epitope, which is carried by subsets of HLA-A and -B allotypes. The A3/11 epitope is carried by the HLA-A*03 and -A*11 allotypes and is recognized by KIR3DL2. Ligands of other activating KIR are poorly characterized in spite of the sequence homology of their ligand-binding extracellular domains to their inhibitory counterparts [13].

Diverse killer Ig-like receptors and their independently segregating HLA ligands constitute a considerable heterogeneity in NK cell responses to targets. Over the last decade, the benefits of NK cell alloreactivity on relapse and relapse-free survival has gained substantial ground in HLA mismatched and haploidentical transplants. However, in HLA matched HCT, these observations have yet to attain consensus. An array of diverse KIR-associated HCT outcomes in the literature owes itself largely to the differences in transplant protocols across various centers, and partly to the ways NK cell alloreactivity is inferred [14, 15]. According to the ‘Perugia model’, the D-R KIR-ligand incompatibility leads to stronger NK cell mediated graft versus leukemia (GVL) effect resulting in protection from leukemia relapse [16, 17]. The ‘Memphis model’ (or the missing ligand model) [18] imparts NK cell alloreactivity to one or more missing HLA ligands in the recipient for the donor inhibitory KIR. The ‘KIR haplotype model’ proposed by the Minnesota group, apportions higher NK alloreactivity to high number of activating KIR genes (haplotype B) in the donor. According to this model, in non-ATG conditioned HCT, use of donors carrying KIR-B haplotypes leads to better overall and relapse-free survival among the recipients carrying one or two C1 bearing HLA-C epitopes. [19–21].

Since several anomalies exist between transplants performed with and without ATG conditioning including incidence of PTLD [22], risk factors for cGVHD [23] and impact of donor CMV status on survival [24] it is conceivable that inferences derived from studies involving transplants performed without ATG conditioning may not be applicable to ATG-conditioned transplants. Therefore, the relevance of the aforementioned KIR-HCT outcome studies in the ATG conditioned and fully HLA-matched HCT seems to be limited, although some of the early studies have demonstrated the need of ATG pre-administration to procure the beneficial effects of NK cells after transplant [6, 25].

Here, we examined the role of all applicable definitions of KIR and KIR-ligands of donor-recipient pairs in HLA matched sibling/unrelated donor HCT using myeloablative conditioning (Fludarabine/Busulfan/ATG with or without TBI).

## Methods

### Subjects and Transplantation

The study cohort consisted of 281 donor (D)/recipient (R) pairs of first allogeneic marrow or blood stem cell transplantation performed in Alberta between December 2004 and December 2012. The study cohort included both sibling (n = 153) and unrelated (n = 128) donors. The D/R pairs were randomly allocated into ‘discovery’ (135 D/R pairs) and ‘validation (146 D/R pairs) cohorts. Patients with engraftment failure, relapse or death prior to 30 days after transplant were excluded. Inclusion criteria were HLA matched (10 out of 10 possible alleles at HLA-A, –B, –C, –DR and –DQ loci), availability of donor-recipient pre-transplant DNA, and at least 2-year follow up for all surviving patients to facilitate evaluation of relapse and cGVHD. All patients received a uniform dose of ATG (4.5 mg/kg) for GVHD prophylaxis and gave written informed consent to participate in the study. Median follow up of surviving patients of the discovery and the validation cohorts was 1746 and 1493 days respectively. The studied population was predominantly Caucasian and most common indications for transplantation were acute myeloid leukemia (37%) followed by acute lymphoblastic leukemia (17%). Clinical and demographic characteristics of D-R pairs are presented in Table 1. The Research Ethics Board of the University of Calgary approved the study.

**Table 1:** Characteristics of Patients, Donors and Transplant.

Characteristic	All cases	Discovery cohort	Validation cohort	p-value*
D-R Pairs (n)	281	135	146	
Median day of follow-up (range)	1280 (31–2632)	1184 (39–2632)	1294 (31–2595)	0.854
Median day of follow-up of surviving patients (range)	1525 (733–2632)	1746 (821–2632)	1493 (733–2595)	0.063
Patient age (yr) at transplantation, median (range)	50 (18–66)	50 (19–66)	50 (18–66)	0.939
Donor age (yr) at transplantation, median (range)	38 (12–68)	39 (12–68)	37 (13–68)	0.863
Donor Gender, Male (%)	181 (64.4)	82 (60.7)	99 (67.8)	0.262
Patient Gender, Male (%)	164 (58.3)	78 (57.7)	86 (58.9)	0.904
Patient age ≥45 at Transplant, n (%)	172 (61.2)	84 (62.2)	88 (60.3)	0.807
Donor age ≥45 at Transplant, n (%)	100 (35.6)	52 (38.5)	48 (32.9)	0.383
Donor Type, Sibling (%); unrelated (%)	153 (54.4); 128 (45.6)	72 (53.3); 63 (46.7)	81 (55.5); 65 (44.5)	0.721
D-R Gender Status at HCT, Male D to Male R (%)	105 (37.3)	44 (32.6)	61 (41.8)	0.139
**Diagnosis, n (%)**^†^				
Lymphoid Malignancies	93 (33.1)	40 (29.6)	53 (36.3)	0.320
Myeloid Malignancies	167 (59.4)	85 (62.9)	82 (56.2)	1.000
Others	21 (7.4)	10 (7.4)	11 (7.5)	1.000
**Stem Cell Source, Peripheral Blood Stem Cells (%)**	271 (96.4)	128 (94.8)	143 (97.9)	0.204
**Disease risk at the time of transplantation, High risk (%)**^‡^	131 (46.6)	58 (42.9)	73 (50.0)	0.282
**Conditioning regimen, n (%)**				
Flu^+^Bu^+^ATG^+^TBI^+^	189 (67.2)	91 (67.4)	98 (67.1)	1.000
Flu^+^Bu^+^ATG^+^TBI^─^	84 (29.9)	38 (28.9)	45 (30.8)	0.355
Other^§^	8 (2.8)	5 (3.7)	3 (2.1)	0.7243
**Donor/Recipient CMV Serostatus, n (%)**				
D+/R+	83 (29.5)	41 (30.3)	42 (28.7)	0.896
D+/R-	28 (9.9)	10 (7.4)	18 (12.3)	0.166
D-/R+	68 (24.2)	33 (24.4)	35 (23.9)	1.000
D-/R-	98 (34.8)	51 (37.7)	47 (32.2)	0.452
Unknown or indeterminate	4 (1.4)	0 (0)	4 (2.7)	
**Acute GVHD by grade, n (%)**				
None	130 (46.2)	56 (41.5)	74 (50.7)	0.149
Grade 1	82 (29.2)	45 (33.3)	37 (25.3)	0.147
Grade 2	42 (14.9)	23 (17.0)	19 (13.0)	0.405
Grade 3/4	27 (9.6)	11 (8.1)	16 (10.9)	0.426
**Chronic GVHD, n (%)**				
None	109 (38.8)	47 (34.8)	63 (43.1)	0.054
Not needing Systemic Therapy (NNST)	35 (12.4)	24 (17.7)	11 (7.5)	0.016
Needing Systemic Therapy (NST)	89 (31.6)	43 (31.8)	45 (30.8)	0.894
Not Evaluable (End of follow-up before day 100)	48 (17.1)	21 (15.5)	27 (18.5)	0.783
**Disease Relapse, n (%)**	61 (21.7)	35 (25.9)	26 (17.8)	0.112
**Median day of onset (range)**				
Acute GVHD Grade II-IV	44 (13–114)	43 (20–99)	49 (13–114)	1.000
Chronic GVHD NST	116 (28–642)	106 (28–465)	131 (83–642)	0.144
Relapse	225 (34–1403)	405 (39–1403)	186 (34–1125)	0.505
Death	246 (31–1605)	293 (39–1465)	208 (31–1605)	0.246
**Cumulative Incidence at the end of follow-up, % (95%CI)**^| |^				
Acute GVHD Grade II-IV	24.5 (19.6.29.7)	24.6 (17.7–32.2)	24.4 (17.7–31.7)	0.936
Chronic GVHD NST	32.9 (27.4–38.6)	33.1 (25.2–41.1)	33.7 (25.6–42.1)	0.774
Relapse	25.2 (19.7–30.9)	26.7 (19.4–35.3)	22.6 (14.9–31.2)	0.786
Death	37.3 (31.2–44.2)	36.1 (28.3–45.1)	40.4 (29.8–53.1)	0.813

Abbreviations: GVHD = graft versus host disease; NST = needing systemic therapy; Flu = fludarabine; Bu = busulfan; ATG = antithymocyte globulin; TBI = total body irradiation.

*Difference in the distribution of patient, donor and transplant characteristics across discovery and validation cohorts estimated by Mann-Whitney Wilcoxon test for patient age, donor age and median days of follow-up; by Gray’s method for cumulative incidences of competing risks data (GVHD and relapse); Kaplan-Meier based log rank test for mortality; and by two-tailed Fisher's Exact test for all other characteristics. p-values <0.05 were considered statistically significant.

^†^Lymphoid malignancies: ALL (Acute Lymphoblastic Leukemia, 20 in discovery cohort and 27 in validation cohort); CLL (Chronic Lymphoblastic Leukemia, 7 in discovery cohort and 10 in validation cohort); NHL (Non-Hodgkin’s Lymphoma, 13 in discovery cohort and 16 in validation cohort) Myeloid malignancies: AML (Acute Myeloid Leukemia, 55 in discovery cohort and 50 in validation cohort); CML (Chronic Myeloid Leukemia, 6 in discovery cohort and 17 in validation cohort); MDS (Myelodysplastic Syndrome, 18 in discovery cohort and 10 in validation cohort).

^‡^Good (or low) risk disease was defined as acute leukemia in first remission, chronic myelogenous leukemia in first chronic or accelerated phase, myelodyplastic syndrome if <5% marrow blasts of aplastic anemia. All other diseases/disease stages were considered high risk.

^§^Cyclophosphamide (Cy) + ATG (4 in discovery cohort and 0 in validation cohort; omitted from analyses of Relapse); Flu+Cy+ATG+TBI (1 in discovery cohort and 1 in validation cohort) or Bu+Cy+ATG (1 in discovery cohort and 1 in validation cohort)

^| |^Determined by Gray’s method for GVHD and relapse and Kaplan-Meier method for estimation of mortality at the end of follow-up.

Conditioning consisted of 250mg/m^2^ Fludarabine (50mg/m^2^/day from day -6 to -2), pharmacokinetically guided ~12.8mg/kg Busulfan (~3.2mg/kg/day from day -5 to -2) and 4.5mg/kg ATG (0.5mg/kg on day -2, 2mg/kg each on days -1 and 0). In addition, total body irradiation (TBI, 2Gy x2, typically on day -1 and day 0) was used in 189 patients (all acute leukemia and some other high risk hematologic malignancies). Additional GVHD prophylaxis included Cyclosporine (starting dose of 2.5mg/kg twice a day intravenously and then targeting plasma levels of 200–400 mg/L) given from day -1 until 3–6 months post-transplant (longer in patients with cGVHD) and Methotrexate given on days +1 (15mg/m^2^), +3, +6 and +11 (10mg/m^2^ each) [3, 26]. Zoster prophylaxis with Acyclovir or Valacyclovir was given for 6–24 months (longer if on immunosuppressive therapy for GVHD). Patients experiencing cytomegalovirus reactivation were preemptively treated with Ganciclovir or Valganciclovir [27].

### Genotyping & models of analyses

The presence or absence of 16 KIR genes was determined by a Luminex^®^-based polymerase chain reaction sequence specific oligonucleotide system (KIRSSO Genotyping Test, One Lambda Inc. and Lifecodes KIR genotyping test, Immucor Inc.) as per manufacturers’ instructions. KIR gene profiles were characterized as per existing definitions of genotypes (AA and B/x) and motifs’ (Cen-A, -B and Tel-A, -B) content and scores (Table 2) [28, 29]. Typing for HLA class-I alleles was obtained from the medical charts. For their association with HCT outcomes, D-R pairs were stratified for analyses as per all applicable definitions. As all D-R pairs were 10/10 HLA matched, (hence KIR-ligand compatible) data were stratified and analyzed for distribution of KIR, KIR-ligand combinations and the missing ligands for donor KIR [14, 30]. In addition to the discovery-validation cohort settings, the data was also separately analyzed in donor-type specific (matched sibling or unrelated) as well as KIR ligand specific settings. Ligand-specific stratification of the study cohort was based on the distribution of C1, C2, Bw4 and A3/11 epitopes of HLA class I antigens in recipients’ genotype. For missing ligand analysis, absence of a KIR ligand (C1, C2, Bw4 or A3/11) in recipient in the presence of putative KIR in donor was scored and analyzed both for individual missing KIR-ligand combinations as well as for a composite absence of one or more missing KIR ligand.

**Table 2:** Description of KIR Genotype Characteristics and their Distribution across Donors and Recipients (All Cases).

KIR Genotype	KIR Genes Present	KIR genes absent	Donor (%)	Recipient (%)
AA	3DL3, 2DL3, 2DP1, 2DL1, 3DP1, 2DL4, 3DL1, 2DS4, 3DL2	2DS2, 2DL2, 2DS3, 2DL5, 3DS1, 2DS5, 2DS1	24.62	31.16
B/x	2DS2, 2DL2, 2DS3, 2DL5, 3DS1, 2DS5, 2DS1		75.38	68.84
Cen-A+	2DL1 and 2DL3		90.15	90.22
Cen-B+	2DS2 and/or 2DL2		58.33	54.71
Tel-A+	3DL1 and 2DS4		94.70	94.93
Tel-B+	3DS1 and/or 2DS1		43.94	42.39
Cen-A/A	2DL1 and 2DL3 only	2DS2 and 2DL2	41.67	45.29
Cen-A/B	2DL3 with 2DS2 and/or 2DL2		48.48	44.93
Cen-B/B	2DS2 and/or 2DL2	2DL3	9.85	9.78
Tel-A/A	3DL1 and 2DS4	3DS1 and 2DS1	56.06	57.61
Tel-A/B	3DL1 and 2DS4 with 3DS1 and/or 2DS1		38.64	37.32
Tel-B/B	3DS1 and/or 2DS1	3DL1 and/or 2DS4	5.30	5.07

Average incidences of individual KIR genes, genotypes and motifs among donors and recipients in both discovery and validation cohorts were consistent with those typically reported among Caucasians [31]. KIR-AA genotype was present in 24.6% donors and 31.1% recipients with remainders representing KIR-B/x genotypes (Table 2).

### Outcomes, competing risks and Statistical analyses

Acute and chronic GVHD were diagnosed as per the National Institutes of Health (NIH) diagnosis and staging working group report [32]. The aGVHD grades were assigned in accordance with the 1994 consensus conference [33] whereas cGVHD was graded as not needing systemic therapy (NNST, usually mild as per NIH criteria) and those needing systemic therapy (NST, usually moderate to severe as per NIH criteria). By aGVHD and cGVHD, the present study henceforth only refers to grade II-IV aGVHD and cGVHD NST respectively. Relapse, death, non-relapse death and relapse-free survival were defined as per standard criteria. cGVHD & relapse-free survival (cGRFS) was defined as a survival free from cGVHD or relapse; in the event of failure, the first event to occur constituted the “failure event” and marked the end of follow-up for that patient. Unlike the recently described GRFS [34], this composite endpoint did not take into account grade III-IV acute GVHD as a separate event. In the present cohort, every incidence of grade III-IV aGVHD was either fatal or preceded cGVHD. As is common in HCT, transplant recipients are subject to multiple potential, both disease related or otherwise, failure outcomes. To evaluate the association of KIR and KIR-ligand repertoires with cause-specific outcomes (GVHD and Relapse), competing risks were taken into account [35, 36]. Graft failure, relapse, second malignancy or death occurring *before* the onset of GVHD was considered competing risk for GVHD. Similarly, graft failure, second malignancy or non-relapse death occurring *before* the onset of relapse was considered competing risk for relapse.

Cumulative incidence function estimates of competing risks data were obtained using the method of Gray [37, 38]. Kaplan-Meier estimation of survivor functions with log rank test was used to estimate cGRFS, RFS and OS. Multivariate analysis of cause-specific outcomes was based on a competing risks regression model of Fine and Gray [35], whereas that of survivor outcomes was based on Cox proportional hazards regression [39]. First, the significance of association of each of the categorical variables (all applicable D-R KIR and KIR-HLA definitions) with HCT outcomes was computed by univariate analysis in the discovery and validation cohorts. Significant associations (p-vales <0.05) were further tested by multivariate analyses with demographic/clinical variables described in literature and/or associated (yielding a p-value ≤0.1) with outcome variables in our univariate analysis (S1 Table) as covariates [23]. Accordingly, covariates for cGVHD were patient age at transplant (≥45y vs. <45y), donor age at transplant (≥45y vs. <45y), donor type (sibling vs. unrelated), D-R gender (M/M vs. others), graft source (PBSC vs. marrow), disease stage (high risk vs. low risk) conditioning regimen (Flu+Bu+ATG with or without TBI), recipient CMV seropositivity (with and without donor CMV serostatus) and aGVHD (gr I-IV) occurring before cGVHD. Covariates for relapse were disease stage, graft source and conditioning regimen. Multivariate analysis for OS and RFS corrected for patient age, disease stage, graft source, CMV serostatus and conditioning, whereas covariates for cGRFS included the ones used for cGVHD and relapse.

All analyses were performed using STATA (Statacorp, College Station TX) and *cmprsk* statistical package of R (www.r-project.org).

## Results

### D-R KIR genotype matching reduces chronic GVHD

At the end of follow-up, the cumulative incidences of aGVHD, cGVHD, and relapse for all studied patients were 24.5% (95%CI: 19.6–29.7%), 32.9% (95%CI: 27.4–38.6%), and 25.2% (95%CI: 19.7–30.9%), respectively (Table 1). KIR genotype mismatching showed a strong correlation with the incidence of cGVHD but not with aGVHD or relapse.

#### (i) Chronic GVHD

**Fig 1:**
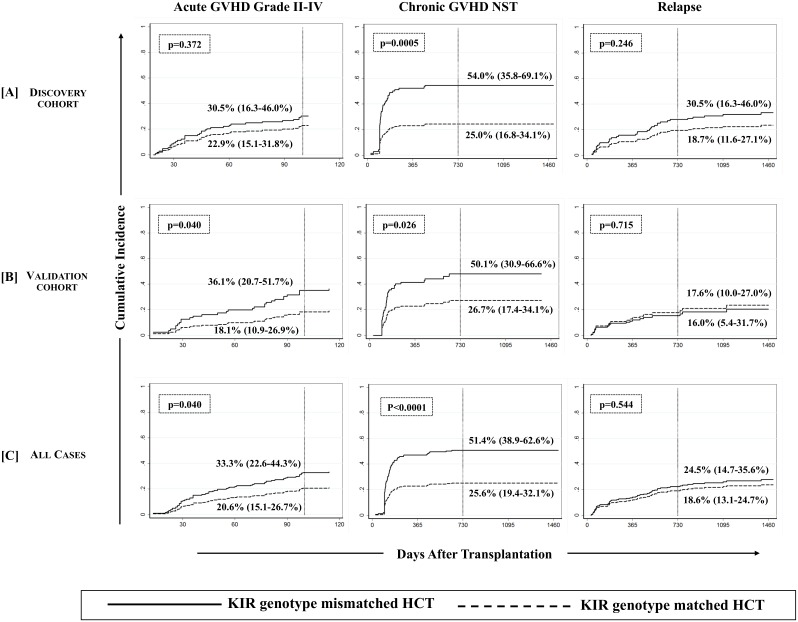
Cumulative incidence function estimates from competing risks data for GVHD and relapse. Cumulative incidences (vertical reference lines) of aGVHD grade II-IV (at day 100 post-transplant, left panel), cGVHD needing systemic therapy (middle panel) and relapse (at 2 years post-transplant, right panel) are presented across [A] discovery, [B] validation and [C] combined cohorts. Gray’s method of cumulative incidence function estimation for competing risks data revealed significantly higher incidences of cGVHD when KIR genotype mismatched donors were used (p-values <0.05 at the end of follow-up).

**Fig 2:**
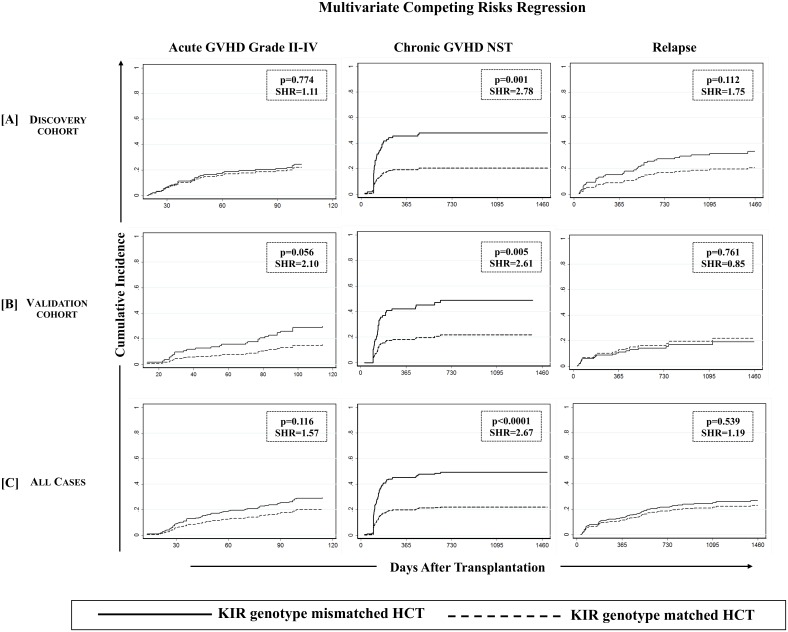
Association of KIR genotype mismatching with chronic GVHD was confirmed using a multivariate competing risks regression model. Effect of KIR genotype mismatching on aGVHD grade II-IV (left panel), cGVHD needing systemic therapy (middle panel) and relapse (right panel) was analyzed using multivariate competing risks regression model (Fine and Gray method) across discovery [A], validation [B] and combined [C] cohorts. Graft failure, relapse, second malignancy or death occurring *before* the onset of cGVHD was considered competing risks for cGVHD. Graft failure, second malignancy or non-relapse death occurring *before* the onset of relapse was considered competing risk for relapse. P values <0.05 were considered statistically significant. Favorable effect of KIR genotype matched donors on cGVHD (middle panel) was confirmed across discovery and validation cohorts in addition to all analyzed cases.

We first analyzed D-R pairs of the discovery cohort to assess the influence of KIR genotypes/matching on different HCT outcomes. A KIR genotype-matched transplant refers to one in which both donor and recipient were either KIR-AA or -B/x type. In univariate analyses, a significantly lower incidence of cGVHD was observed with D-R KIR genotype matched transplants compared to the KIR genotype-mismatched (B/x donor into AA recipients or vice-versa) transplants of the discovery cohort (25.0% [95%CI: 16.8–34.1%] vs. 54.0% [95%CI: 35.8–69.1%], p = 0.0005; Fig 1A, middle panel). Analyses of validation cohort further confirmed the observations as the incidence of cGVHD was lower among KIR genotype matched transplants compared to KIR-genotype mismatched transplants (26.7% [95%CI: 17.4–34.1%] vs. 50.1% [95%CI: 30.9–66.6%], p = 0.026, Fig 1B, middle panel). The multivariate competing risks regression analysis established a strong association of KIR genotype mismatching with cGVHD in patients of both discovery (p = 0.001, Table 3, Fig 2A, middle panel) and validation cohorts (p = 0.005, Table 3, Fig 2B, middle panel). Upon combining discovery and validation cohorts, the cumulative incidence of cGVHD in KIR genotype-matched versus mismatched transplants presented a sub-distributional hazard ratio of 2.67 (p<0.0001; Fig 2C, middle panel). Favorable cGVHD outcome through KIR genotype matching was contributed by both KIR-B/x and –AA genotype matching (p = 0.011 and 0.009 respectively, Table 3) and was applicable to both matched sibling and unrelated donor transplants (p = 0.009 and 0.004 respectively, Table 3).

**Table 3:** Association of Donor-Recipient KIR Genotype Mismatching with the Outcomes of Allogeneic HCT.

		Acute GVHD Grade II-IV			Chronic GVHD NST			Relapse		
Marker	N	*p*	SHR	95%CI	*p*	SHR	95%CI	*p*	SHR	95%CI
***Overall D-R KIR Genotype Mismatching (B/x-Donor to AA-Recipient or vice-versa)***										
Discovery cohort	36/136	0.774	1.11	0.52–2.35	**0.001**	**2.78**	**1.50–5.17**	0.112	1.75	0.87–3.51
Validation Cohort	43/145	0.056	2.10	0.98–4.52	**0.005**	**2.61**	**1.33–5.11**	0.761	0.85	0.31–2.33
All Cases	79/281	0.116	1.57	0.89–2.63	**<0.0001**	**2.67**	**1.70–4.19**	0.539	1.19	0.68–2.08
***Donor Type (All cases)***										
Matched Sibling Donors	32/153	0.408	1.41	0.62–3.21	**0.009**	**2.29**	**1.23–4.29**	0.596	1.24	0.55–2.77
Matched Unrelated Donors	47/128	0.190	1.67	0.77–3.62	**0.004**	**2.62**	**1.37–5.01**	0.844	1.08	0.48–2.44
***KIR-Ligand Type (All cases)***										
D-R HLA-A*03/A*11 positive	26/117	0.849	1.10	0.40–3.03	**0.004**	**3.03**	**1.43–6.44**	0.324	1.57	0.64–3.87
D-R HLA-A*03/A*11 negative	53/164	0.208	1.62	0.76–3.46	**0.018**	**2.29**	**1.15–4.54**	0.746	1.14	0.52–2.48
D-R HLA Bw4/x	45/159	0.313	1.45	0.70–3.01	**0.013**	**2.19**	**1.18–4.05**	0.303	1.51	0.68–3.30
D-R HLA Bw6/6	34/122	0.594	1.29	0.50–3.32	**0.003**	**3.11**	**1.45–6.65**	0.791	1.12	0.48–2.56
D-R HLA-C group C1/x	67/237	0.243	1.43	0.78–2.64	**0.001**	**2.40**	**1.42–4.06**	0.785	1.09	0.58–2.11
D-R HLA-C group C2/2	12/44	0.667	1.41	0.29–6.91	0.937	1.11	0.08–14.73	0.165	2.73	0.76–10.97
***KIR Genotype Specific Matching (All Cases)***										
B/x Donor to AA Recipient	49/211	0.301	1.41	0.73–2.74	**0.011**	**2.13**	**1.19–3.81**	0.865	1.06	0.52–2.07
AA Donor to B/x Recipient	30/70	0.293	1.98	0.55–7.15	**0.009**	**3.80**	**1.39–10.38**	0.252	1.99	0.61–6.51

Abbreviations: GVHD = Graft versus host disease; NST = needing systemic therapy; SHR = sub hazard ratio (sub-distributional hazard); D = donor; R = recipient; N = number of observations (KIR genotype mismatching) out of number of recipients in the corresponding cohort

A multivariate competing risks regression model was used to estimate the effect of donor-recipient mismatching for KIR genotypes on GVHD (acute and chronic) and relapse across discovery and validation cohorts individually and in combination (all cases). The overall cohort (all cases) was also classified into donor type (matched sibling or unrelated) as well as ligand specific cohorts. Ligand specific cohort stratification was based on the presence and absence of four polymorphic HLA class-I epitopes (A3/A11, Bw4, C1 and C2), which constitute ligands for KIR. A combined group of Bw4/4 and Bw4/6 was designated as Bw4/x, whereas the combined group of C1/C1 and C1/C2 recipients was designated as C1/x. Significance of association or KIR genotype matching with HCT outcomes was separately tested across donor-type and ligand-specific cohorts using the multivariate competing risks regression model. Sub-distributional hazard were described as sub-hazard ratios (SHR); p-values <0.05 were considered statistically significant (presented here in bold fonts).

**Fig 3:**
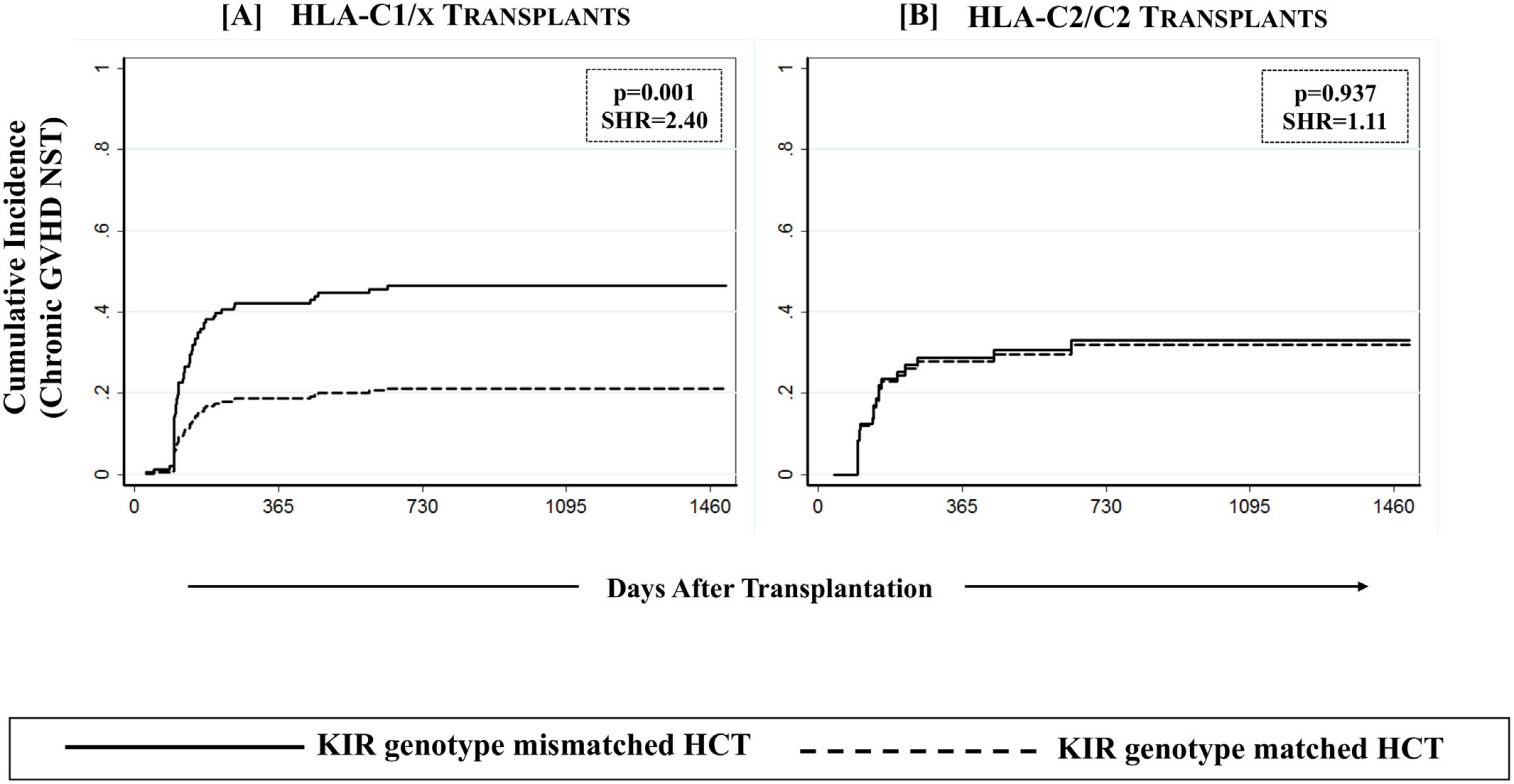
Effect of KIR genotype matched donors on cGVHD among recipients having one or more C1 bearing HLA-C epitopes. Effect of KIR genotype matching was analyzed in a combined group of C1/C1 and C1/C2 recipients (designated as C1/x) as well as in C2/C2 homozygote recipients using a multivariate competing risks regression model.

The effect of KIR genotype matching on cGVHD was further assessed in a KIR-ligand specific setting where the overall study cohort was stratified on the basis of the presence/absence of the four polymorphic epitopes of HLA class-I alleles that constitute ligands for various KIR. Though none of the HLA class-I epitopes by themselves influenced the outcomes of HCT (data not shown), when analyzed in conjunction with KIR genotype matching in a preliminary analysis, the effects of C1 predominated those of C2. The combined group of C1/C1 and C1/C2 recipients was therefore designated as C1/x and was compared with C2/C2 homozygotes. Combined group of Bw4/4 and Bw4/6 was designated as Bw4/x and was compared with Bw6/6. Similarly A*03/A*11 carriers were compared with A3/11 epitope negatives. The beneficial association of KIR genotype matching with cGVHD was specific to recipients having one or more C1 bearing HLA-C epitopes (C1/x; p = 0.001, Table 3, Fig 3A). There was no effect of KIR genotype matching on cGVHD among recipients having two C2 epitopes (C2/C2; p = 0.937, Table 3, Fig 3B). Stratification of the overall cohort based on either A3/11 or Bw4 epitopes did not segregate the effects of KIR genotype matching on cGVHD (Table 3).

#### (ii) Acute GVHD

Although KIR-genotype mismatching was associated with an increased incidence of aGVHD in the univariate analysis (p = 0.040, among all analyzed cases, Fig 1C, left panel), this effect was not significant in the multivariate competing risks regression analysis in each of discovery-validation (Table 3, Fig 2A–2C, left panel), donor-type specific or ligand-specific cohorts (Table 3)

None of the other reported definitions pertinent to either KIR alone or in combination with HLA class-I yielded a significant association with either cGVHD or aGVHD (S2 Table).

#### (iii) Relapse

Unlike other treatment strategies, a reduced risk of cGVHD among KIR genotype matched HCT was not found to be associated with an increased risk of relapse in each of discovery-validation (Table 3, Fig 2A–2C, right panel), donor-type specific or ligand-specific cohorts (Table 3). Interestingly, in a time-to-event analysis on all studied cases, the 2 year post-transplant cumulative incidence of relapse fared comparatively better in KIR matched (18.6% [95%CI: 13.1–24.7%]) than in KIR mismatched (24.5% [95%CI: 14.7–35.6%]) transplants, although this difference was not statistically significant at the end of follow up (p = 0.544 Fig 1C, right panel). This effect did not segregate as per the diagnosis or primary disease for which transplant was performed. There was no statistically significant difference in the incidence of relapse between lymphoid and myeloid malignancies with or without taking KIR genotype matching into consideration (data not shown).

### Missing inhibitory KIR Ligands protect against relapse

**Fig 4:**
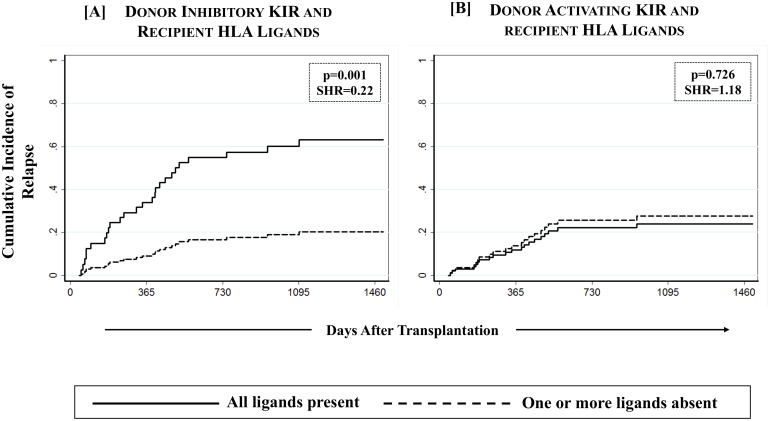
Effect of missing HLA ligands in recipients of unrelated donor HCT. Absence of one or more missing HLA ligands was scored for donor inhibitory KIR3DL2 (A3/A11), KIR3DL1 (Bw4), KIR2DL2/2DL3 (C2) and KIR2DL1 (C1) as well as activating KIR2DS1 (C2) and KIR3DS1 (Bw4). A multivariate competing risks regression analysis revealed a significantly reduced incidence of relapse (p-values <0.05) among recipients of unrelated donor HCT in which one or more HLA ligands for donor inhibitory KIR were absent.

Among the various definitions of NK cell alloreactivity based on donor KIR and recipient HLA combinations, only ‘missing ligand model’ is applicable to HLA matched transplants [14]. In this model, the absence of a putative HLA ligand in the presence of its receptor in donor NK cells (missing ligand) mimics what would be a ‘missing self’ scenario in an individual, and would incite a response to target cell (in this case, residual malignant cells). Beneficial effects of alloreactive NK cells in reducing relapse and improving a progression free survival have been reported in a variety of transplant settings [14–17, 25, 40]. To this end, we tested the effects of missing A3/11 (KIR3DL2 ligand), Bw4 (KIR3DL1/3DS1 ligand), C1 (KIR2DL2/2DL3 ligand) and C2 (KIR2DL1/2DS1 ligand) in recipients when the corresponding receptors were present in their respective donors. A significantly reduced incidence of relapse was observed in recipients of unrelated donor transplants who were missing one or more inhibitory KIR ligands as compared to those in which all inhibitory KIR ligand(s) were present (21.6% [13.7–30.6%] vs. 63.6% [13.5–90.3%]; p = 0.001, Table 4, Fig 4A). However, similar effect was not seen in the recipients of sibling donor transplants. None of the individual donor-KIR missing-HLA combination yielded any notable effect (S2 Table), nor was there a dose effect with the number of missing KIR ligands. When a separate analysis with data stratified as per disease type (lymphoid vs. myeloid) was conducted, the effect of missing inhibitory KIR ligands on relapse did not segregate according to the type of primary disease (data not shown).

**Table 4:** Effect of Missing KIR Ligands on the Outcomes of Allogeneic HCT.

		Acute GVHD Grade II-IV			Chronic GVHD NST			Relapse		
Marker	N	*p*	SHR	95%CI	*p*	SHR	95%CI	*p*	SHR	95%CI
***One or more missing ligands for donor inhibitory KIR***										
Discovery cohort	117/136	0.524	0.69	0.22–2.14	0.753	1.18	0.42–3.21	0.629	0.80	0.33–1.96
Validation Cohort	133/145	0.992	0.99	0.22–4.43	0.575	0.67	0.17–2.66	0.443	2.12	0.31–4.49
All Cases	250/281	0.725	0.85	0.35–2.05	0.740	1.14	0.52–2.49	0.955	0.98	0.45–2.14
***Donor Type***										
Matched Sibling Donors	133/153	0.726	0.78	0.21–3.01	0.670	0.82	0.33–2.02	0.100	5.66	0.73–43.78
Matched Unrelated Donors	116/128	0.820	1.22	0.21–7.02	0.314	2.27	0.45–11.29	**0.001**	**0.22**	**0.10–0.54**
***One or more missing ligands for donor activating KIR***										
Discovery cohort	39/91	0.763	0.87	0.35–2.15	0.498	0.75	0.33–1.68	0.444	0.74	0.34–1.61
Validation Cohort	41/86	0.736	1.161	0.48–2.82	0.439	0.73	0.32–1.61	0.276	1.69	0.66–4.34
All Cases	80/177	0.965	0.98	0.53–1.82	0.364	0.77	0.44–1.34	0.879	1.05	0.57–1.92
***Donor Type***										
Matched Sibling Donors	45/91	-	-	-	0.673	0.83	0.35–1.96	0.917	0.95	0.42–2.17
Matched Unrelated Donors	35/86	0.688	1.21	0.47–3.08	0.654	0.79	0.30–2.12	0.726	1.18	0.46–3.01

Abbreviations: GVHD = Graft versus host disease; NST = needing systemic therapy; SHR = sub hazard ratio (sub-distributional hazard); N = number of observations (missing KIR ligands) out of number of recipients in the corresponding cohort; “-” denotes distributions in which statistical computation was not possible.

Missing ligands for inhibitory KIR included absence of at least one of A3/11, Bw4 (KIR3DL1 ligand), C1 (KIR2DL2/2DL3 ligand) or C2 (KIR2DL1 ligand) in recipients when the corresponding inhibitory receptors were present in their respective donors. Missing ligands for activating KIR included absence of either Bw4 (KIR3DS1 ligand) or C2 (KIR2DS1 ligand) in recipients when the corresponding activating receptors were present in the donor. Other poorly characterized activating KIR-ligand combinations, though dispersed in literature, were excluded from this analysis but were taken into consideration in supplementary data. A multivariate competing risks regression model was used to estimate the effect missing ligands on GVHD (acute and chronic) and relapse across discovery and validation cohorts individually and in combination (all cases). Significance of association was also tested separately among recipients of sibling and unrelated donor HCT. Sub-distributional hazard were described as sub-hazard ratios (SHR); p-values <0.05 were considered statistically significant (presented here in bold fonts).

### KIR genotype matched donors and missing inhibitory KIR ligands in recipients differentially affect survival outcomes

For all studied patients, relapse free survival (RFS), cGVHD & relapse free survival (cGRFS) and overall survival (OS) at the end of follow-up was 57.3% (95%CI: 50.5–63.6%), 33.6% (95%CI: 27.6–39.7%) and 62.6% (95%CI: 55.7–68.7%) respectively. None of the tested KIR and KIR-HLA definitions showed any significant effect on OS but HCT recipients with KIR genotype matched donors had significantly better cGRFS, while unrelated HCT recipients missing one or more ligands for donor KIR had improved RFS.

**Fig 5:**
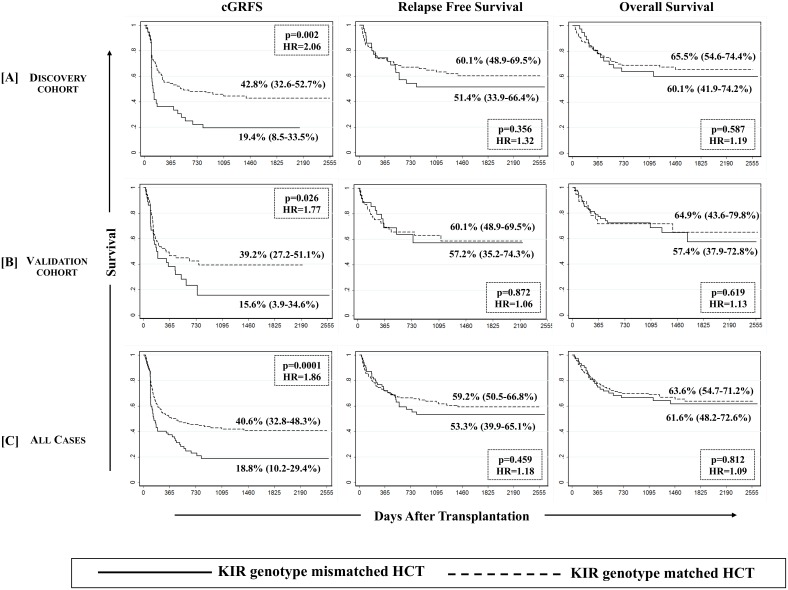
Effect of KIR genotype matching on the survival outcomes. Estimates of cGVHD & relapse free survival (cGRFS, left panel), relapse-free survival (RFS, middle panel) and overall survival (OS, right panel) are presented across discovery [A], validation [B] and combined [C] cohorts. A multivariate Cox Proportional Hazards Regression at the end of follow-up revealed a significantly poorer (p-values <0.05) cGRFS in both discovery and validation cohorts in addition to a combined cohort (discovery + validation) when KIR genotype mismatched donors were used.

**Fig 6:**
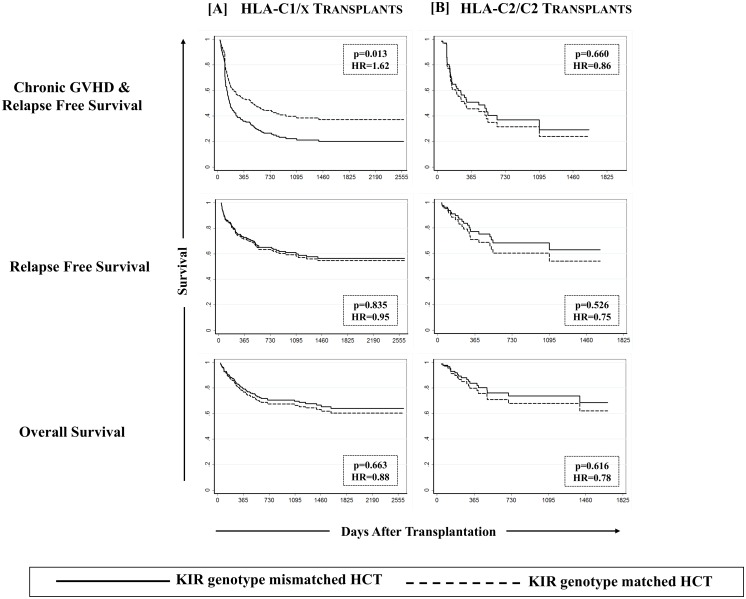
Effect of KIR genotype matching on cGVHD & relapse-free survival (cGRFS) among C1/x recipients. As with cGVHD, KIR genotype matching segregated only the recipients having one or two C1 bearing HLA-C allotypes for differences in cGRFS in a multivariate Cox proportional hazards regression that accounted for covariates for both GVHD and relapse. No effect on relapse free survival (RFS) or overall survival (OS) was noted.

Chronic GVHD & relapse free survival (cGRFS) was defined as one being free of cGVHD or relapse; whichever happened first, constituted the “failure event” and marked the end of follow-up for that patient. Multivariate analysis performed for cGRFS corrected for covariates for both GVHD and relapse showed that cGRFS is significantly improved in recipients of KIR genotype-matched transplants as compared to the recipients of KIR genotype-mismatched transplants in both discovery and validation cohorts (p = 0.002 and 0.026 respectively; Fig 5A and 5B, left panel). Upon combining the discovery and validation cohorts, a hazard ratio of 1.86 (95%CI: 1.32–2.59, p = 0.0001) was observed, where cGRFS was significantly lower in recipients of KIR genotype mismatched transplant as compared to those of KIR genotype matched transplant (18.8% [95%CI: 10.2–29.4%] vs. 40.6% [95%CI: 32.8–48.3%]; Fig 5C, left panel). None of the other tested definitions yielded any significant effect on cGRFS (data not shown). When a similar analysis was performed in ligand-specific cohort, the favorable cGRFS was observed exclusively in recipients carrying one or two C1 bearing HLA-C allotypes (p = 0.013, Fig 6A), receiving grafts from a KIR genotype matched donor. Matched sibling or unrelated donors, Bw4/x positive or negative recipients or A3/11 positive or negative recipients did not segregate the beneficiaries of KIR genotype matching in terms of improved cGRFS.

**Fig 7:**
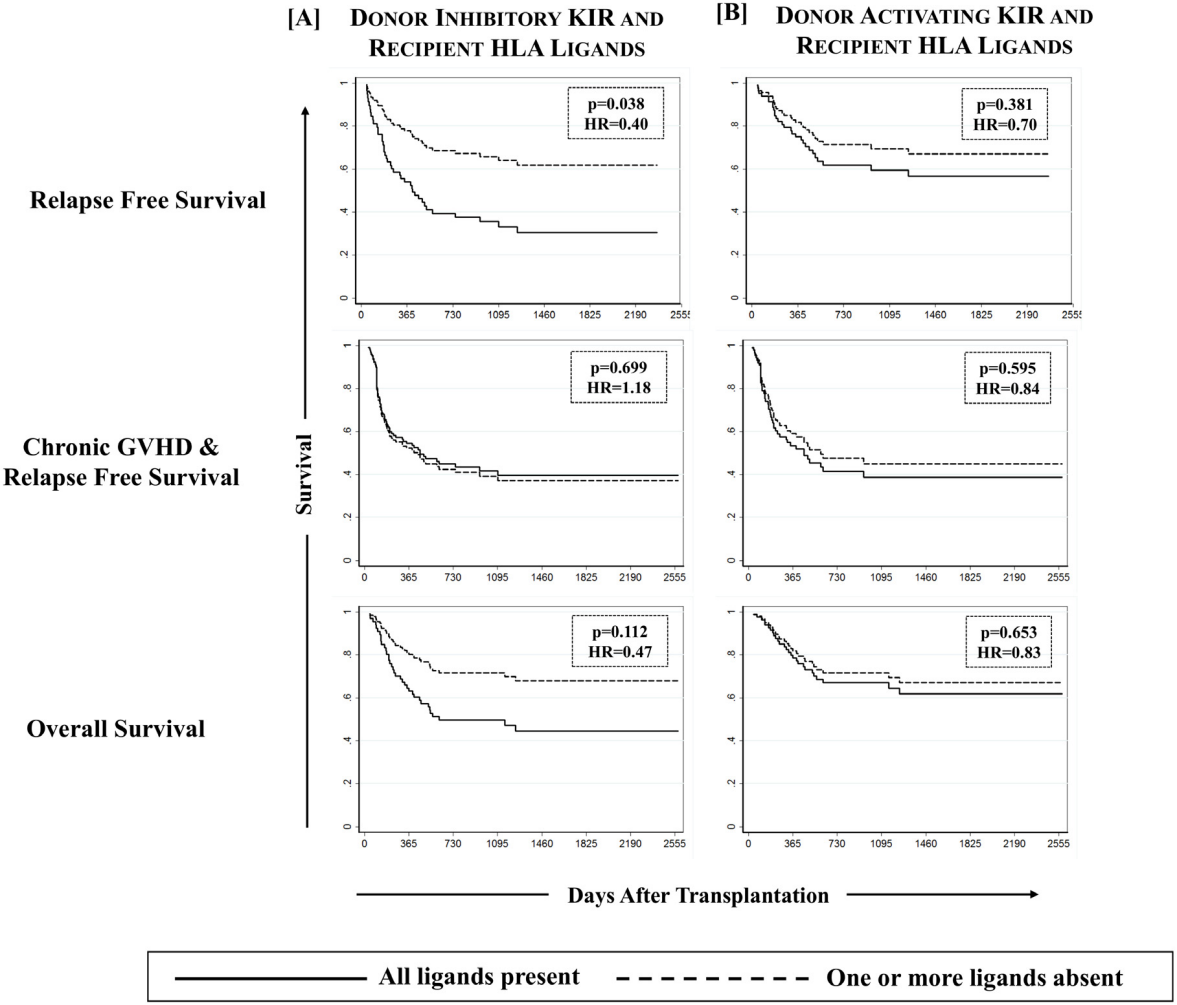
Effect of missing KIR ligands on relapse free survival among recipients of unrelated donor HCT. Multivariate cox proportional hazards regression analysis revealed a significantly improved relapse free survival (p-values <0.05) among recipients of unrelated donor HCT in which one or more HLA ligands for donor inhibitory KIR were absent. No effect on cGVHD & relapse-free survival (cGRFS) was noted whereas recipients with missing inhibitory KIR ligands experienced a comparatively better overall survival (OS), the difference however was not significant.

As expected, unrelated donor HCT recipients missing one or more ligands for donor inhibitory KIR had a significantly improved RFS as compared to the HCT recipients in which all KIR ligands were present (61.9% [50.3–71.6%] vs. 20.0% [1.4–54.7%]; p = 0.038, Fig 7A). No notable beneficial or adverse effect of KIR genotype matching was observed on RFS among discovery and validation cohorts (Fig 5) as well as when the cohort was stratified according to donor type or ligand type (Fig 6).

Although, none of tested KIR and KIR-HLA definitions had any impact of overall survival, a comparatively better overall survival was observed among recipients of unrelated donor HCT who were missing one or more ligands for donor inhibitory KIR as compared to those having all the ligands present, although this difference was not statistically significant at the end of follow up (67.5% [95%CI: 56.2–76.5%] vs. 42.4% [95%CI: 13.7–69.1%], p = 0.112, Fig 7A).

## Discussion

The present study examined the effects of donor and recipient KIR and HLA class-I types across all existing definitions of KIR and KIR-HLA combinatorial diversity in HLA matched HCT following T cell depleted myeloablative conditioning. Observed associations were tested in a discovery-validation setting in addition to donor type (matched sibling or unrelated) as well as ligand-specific settings. First, we discovered (and subsequently validated) that D-R matching for KIR genotypes reduced chronic GVHD requiring systemic therapy and improved survival free of chronic GVHD and relapse (cGRFS). This effect was applicable to both sibling and unrelated donor HCT and was specific to recipients carrying one or two C1 bearing HLA-C allotypes. Second, absence of one or more of recipient A3/11, Bw4, C1 or C2 ligands in the presence of their putative donor inhibitory KIR (missing ligand) significantly reduced relapse and improved relapse-free survival (RFS) when an unrelated donor was used. Finally, none of the benefits of other reported definitions pertaining to KIR alone or in combination with HLA class-I of donors and recipients were found relevant in the current setting.

These observations, though not directly tested for functional mechanisms in vitro, draw parallels from several published reports by us and others which are suggestive of a possible role of NK cells in controlling GVHD, especially when transplant conditioning includes ATG administration. First, we have shown that ATG pre-administration for in vivo T cell depletion (aimed at GVHD prophylaxis) favors a rapid quantitative reconstitution of NK cells [41]. A low NK:T cell ratio in the graft may favor T-cell interaction with host antigen presenting cells (APC) thereby initiating GVHD, whereas a high NK:T cell ratio may favor APC elimination hence preventing GVHD [14, 42]. Early reconstitution of NK cells is marked by a fast increase in the CD56^bright^ fraction with a relatively slower recovery of CD56^dim^ fraction [41, 43], and an increased risk of cGVHD with lower counts of CD56^bright^ NK cells ~3 months after ATG-conditioned HCT was observed [44]. Further, in a recent study we reported that high serum IL-15 levels on day +7 were associated with a lower incidence of cGVHD [45]. Although these levels did not correlate with improved quantitative reconstitution of NK cells in this study (measured at day +28), IL-15 has been shown to restore functions of NK cells that show low cytotoxicity and diminished IFN-γ production up to 3 months after HCT [46].

Approximately 15–19% of HCT recipients are C2/C2 homozygotes and some studies have implicated the absence of C1 epitope with poorer HCT outcomes [21, 30, 47, 48]. However in our experience, similar incidences were observed among C1/x recipients as compared to C2/C2 homozygotes, both in terms of relapse (26.6% [20.1–33.5%] vs. 28.7% [13.1–46.5%], p = 0.597) as well as cGVHD (33.3% [26.9–39.8%] vs. 37.2% [19.9–54.6%], p = 0.774). These differences can be attributed to the pre-administration of ATG for in vivo T cell depletion to all patients prior to transplant. Used as an effective GVHD prophylactic drug, the polyclonal ATG contains antibodies recognizing multiple surface markers, some of which are expressed on leukemic cells [49] and has been shown to cause lysis of leukemic cell lines [50] as well as leukemic blasts in vitro [51]. Nevertheless, the favorable effect of KIR genotype matched donors on cGVHD as well as on cGRFS being applicable only to C1/x recipients seems to be consistent with C2 self-ligand rendering NK cells expressing its putative receptors (KIR2DL1/2DS1) non-responsive as hypothesized in some of the earlier studies [21, 30].

A significant reduction in the incidences of cGVHD with KIR genotype matched donors did not accompany a heightened risk of relapse, which was unexpected, and highlighted a critical facet of this algorithm. NK cells’ discrimination of healthy from malignant cells despite lacking antigen specificity separates GVL from GVHD. Since normal cells do not express the required amounts of activating ligands to incite an NK cell response, NK cells with matched KIR genotypes may simultaneously be controlling GVHD. ATG facilitated mechanisms including rapid NK-cell quantitative and functional reconstitution [41] and suppression of residual T-cell alloreactivity by NK cells [52, 53] further support these observations. Alternatively, as suggested by at least one recent clinical trial pertaining to activated NK cell DLI, NK cells may either be exacerbating otherwise subclinical T-cell mediated alloreactivity or directly killing tissues expressing activating receptor ligands, contributing to, rather than preventing GVHD [54].

Consistent with other published reports [55, 56], and the hypothesis that donor derived NK cells would mimic ‘missing-self’ when the recipient is missing ligand for donor KIR, a significant favorable effect on relapse and relapse free survival was observed with one or more missing inhibitory KIR ligand when an unrelated donor was used. No such effect was observed in HLA identical sibling donor transplants, which is consistent with the existing literature exploring missing ligand model primarily in unrelated donor HCT. Recipients with one or more missing ligands for donor inhibitory KIR had a comparatively better overall survival (67.5% vs. 42.7% when all ligands were present), but this difference did not reach statistical significance. No effect of any of the tested KIR-HLA definitions was noted on severe (grade II-IV) acute GVHD, which could be due to the predominant influence of in vivo T cell depletion using ATG on early than late post-transplant events and variability in reconstitution of mature NK cell receptor repertoire after HCT [57–59].

Interest in KIR-HLA-regulated NK cells and their influence on HCT outcomes has led to many reports in HLA matched and mismatched transplants with diverse, often difficult to integrate, outcomes. A series of studies reported beneficial effects of KIR B/x genotype donors consisting of centromeric-B motifs in controlling relapse among recipients carrying at least one C1 bearing HLA-C allotypes predominantly in HLA-C mismatched transplants [19–21]. Other studies have reported beneficial and adverse clinical effects of donor activating KIR genes [60–65] or their incompatibilities with recipients’ genes [6, 66] in different transplant settings. However, in this study, we did not find any effect of donor KIR-B/x (or KIR-AA) genotypes on any of the studied clinical end points, both when tested within broad definitions, and when analyzed in terms of centromeric and telomeric B-motifs. The present study is unique from other published reports in the inclusion of ATG pre-administered, 10/10 HLA allele matched transplants. In a setting of HLA-C ligand mismatched, T-cell replete, unrelated donor transplant, Yabe et al noted that the beneficial effects of KIR-regulated NK cells depended on ATG pre-administration [25].

There could be certain limitations to this study. First, the studied cohort is heterogeneous with respect to diagnosis, but is homogenous in terms of pre-transplant conditioning. However in a separate analysis the observed associations were not affected by the types of primary disease for which HCT were performed making the findings applicable to and representing all (lymphoid and myeloid) hematologic malignancies. Second, our novel description of cGRFS excludes aGVHD grade III-IV as compared to the Minnesota group’s definition of GVHD and relapse-free survival (GRFS) [34]. In the present study, all patients experiencing grade III-IV aGVHD either died or developed cGVHD, yielding similar observations when tested for KIR genotype matching and GRFS. Furthermore, chronic GVHD, apart from rarely being a de novo development, in most cases may either be an extension of acute GVHD or may emerge after a quiescent interval after acute GVHD is resolved. Multivariate analyses for both cGVHD and cGRFS therefore corrected for the ‘occurrence of aGVHD (grade I-IV) before cGVHD’. Third, the studied cohort includes a substantial number of sibling donors, who are more likely to be matched for KIR genotypes as compared to unrelated donors. To rule out the possibility that KIR genotype matching is merely a marker of relatedness, sibling donors were accounted for in the multivariate analyses in discovery-validation and ligand-specific settings in addition to being separately tested for observed associations in a donor-type specific setting. Fourth, the observed correlations were made through genetic analysis only and a functional mechanism attributable to the beneficial effects of KIR genotype matching remains poorly understood. Nevertheless, robust statistical modeling and cohort stratification rule out erroneous associations and as a result the study offers a clinically applicable donor selection strategy that can help further control cGVHD without affecting the risk of disease relapse and/or identify patients at high risk of developing cGVHD or relapse as potential candidates for preemptive or prompt therapies.

In conclusion, the present study for the first time presents the beneficial effects of KIR genotype matching in an ATG conditioned myeloablative HLA matched transplant setting. Worthy of note, our observations assert that the lessons learnt pertaining to the previously understood effects of KIR and KIR-HLA combinatorial diversity in utilizing NK cells to the benefit of HCT outcomes had limited relevance in an ATG conditioned HLA matched HCT setting as evidenced by previously unreported findings and the assimilation of supplementary table (S2 Table). Findings of this study have the potential to further augment the beneficial outcomes of HCT using similar pre-transplant regimen [67, 68]. We have estimated the practical applicability of suggested KIR genotype matched donor selection strategy by adding an inexpensive KIR genotyping (available in most HLA laboratories), which can be used today to supplement HLA typing. By KIR genotyping three HLA matched donors during confirmatory HLA typing (currently one of two HLA matched unrelated donors are selected) the probability of finding a KIR genotype matched donor will increase from current random rate of 64% to an estimated >90% that will result in at least 12–15% reduction in the incidence of cGVHD and ~10% increase in survival free of cGVHD and relapse. Accrual for a prospective clinical trial selecting the HLA matched donors who are also matched for KIR genotypes is expected to start by early 2017.

## Supporting Information

S1 Table Univariate analyses for the association of Demographic and Clinical Variables with the HCT outcomes. (DOCX)

S2 Table KIR/KIR-HLA definitions yielding no association with HCT outcomes. (DOCX)
