# Supplementary material for: Donor-Recipient Matching for KIR Genotypes Reduces Chronic GVHD and Missing Inhibitory KIR Ligands Protect against Relapse after Myeloablative, HLA Matched Hematopoietic Cell Transplantation
Source: PLoS One. 2016 Jun 24;11(6):e0158242. doi: 10.1371/journal.pone.0158242 (PMC4920429; doi:10.1371/journal.pone.0158242)
Supplement: S1 Table — (DOCX) [file pone.0158242.s001.docx]

**Table S1: Univariate analyses for the association of Demographic and Clinical Variables with the HCT outcomes**

| **Demographic/Clinical variables (n=281 D-R pairs)** | **Distribution** | **Gr II-IV aGVHD** | **cGVHD NST** | **Relapse** | **RFS** | **CGRFS** | **OS** |
| --- | --- | --- | --- | --- | --- | --- | --- |
| **Patient age, years** | ≥45 = 172; <45 = 109 | 0.139 | **0.039** | 0.564 | 0.105 | 1.000 | 0.134 |
| **Donor age, years** | ≥45 = 100; <45 = 181 | 0.518 | 0.209 | 0.573 | 0.206 | 0.632 | 0.248 |
| **Donor Type** | Sibling = 153; Unrelated = 128 | 0.098 | 0.623 | 0.754 | 0.376 | 0.301 | 0.495 |
| **Donor (D)-Recipient (R) Gender** | M/M = 105; Others = 176 | 0.121 | 0.315 | 0.840 | 0.822 | 0.169 | 0.802 |
| **Graft Source** | PBSC = 271; BM = 10 | 0.679 | 0.712 | 0.300 | 0.121 | 0.308 | 0.235 |
| **Disease Stage** | High risk= 131; Low Risk=150 | 0.215 | 0.145 | **0.045** | **0.035** | **0.002** | 0.195 |
| **Conditioning regimen** | Flu+Bu+ATG+TBI= 189; Others = 92 | 0.110 | **0.0001** | 0.169 | 0.324 | **0.004** | 0.227 |
| **Recipient CMV Serostatus** | R+ = 151; R- = 126 | 0.360 | 0.881 | 0.542 | 0.343 | 0.317 | 0.134 |
| **Donor-Recipient CMV Serostatus** | D+R+ = 83; Other = 194 | 0.932 | 0.679 | 0.469 | 0.449 | 0.921 | 0.443 |
|  | D+R- = 28; Other = 249 | 0.388 | 0.358 | 0.879 | 0.765 | 0.512 | 0.284 |
|  | D-R+ = 68; Other = 209 | 0.158 | 0.931 | 0.945 | 0.065 | 0.293 | **0.013** |
|  | D-R- = 98; Other = 179 | 0.429 | 0.903 | 0.587 | 0.421 | 0.534 | 0.359 |
| **aGVHD before cGVHD** | Yes = 59; No = 30 | NA | **0.002** | NA | NA | **0.006** | 0.100 |

Abbreviations: aGVHD = acute GVHD; cGVHD = chronic GVHD; RFS = Relapse-free survival; CGRFS = chronic GVHD and relapse free survival; OS = overall survival; M = male; PBSC = peripheral blood stem cells; BM = bone marrow; CMV = cytomegalovirus; NA = Not applicable.

Effect of clinical and demographic characteristics on GVHD (acute and chronic) and relapse were estimated by univariate competing risks regression analysis and on survival (RFS, CGRFS and OS) by Kaplan-Meier based log-rank test. p-values <0.05 were considered statistically significant (presented here in bold fonts).
