# Supplementary material for: Donor-Recipient Matching for KIR Genotypes Reduces Chronic GVHD and Missing Inhibitory KIR Ligands Protect against Relapse after Myeloablative, HLA Matched Hematopoietic Cell Transplantation
Source: PLoS One. 2016 Jun 24;11(6):e0158242. doi: 10.1371/journal.pone.0158242 (PMC4920429; doi:10.1371/journal.pone.0158242)
Supplement: S2 Table — (DOCX) [file pone.0158242.s002.docx]

**Table S2: KIR/KIR-HLA definitions yielding no association with HCT outcomes**

| **Marker** | **N** | **Grade II-IV Acute GVHD** | | | **Chronic GVHD NST** | | | **Relapse** | | |
| --- | --- | --- | --- | --- | --- | --- | --- | --- | --- | --- |
|  |  | ***p*** | **SHR** | **95% CI** | ***p*** | **SHR** | **95% CI** | ***p*** | **SHR** | **95% CI** |
| ***KIR Models (1-8)*** |  |  |  |  |  |  |  |  |  |  |
| **Donor B/x positive** |  |  |  |  |  |  |  |  |  |  |
| *Matched sibling donors* | 118/153 | 0.219 | 2.01 | 0.66-6.07 | 0.528 | 0.78 | 0.36-1.68 | 0.624 | 1.25 | 0.51-3.07 |
| *Matched unrelated donors* | 93/128 | 0.758 | 1.13 | 0.51-2.50 | 0.243 | 0.65 | 0.32-1.33 | 0.838 | 1.10 | 0.43-2.79 |
| *HLA-A*03/-A*11 positive* | 89/117 | 0.455 | 1.50 | 0.52-4.37 | 0.929 | 0.96 | 0.39-2.32 | 0.130 | 0.51 | 0.21-1.22 |
| *HLA-A*03/-A*11 negative* | 122/164 | 0.188 | 1.93 | 0.72-5.11 | 0.339 | 0.71 | 0.34-1.44 | 0.060 | 4.33 | 0.97-19.42 |
| *HLA Bw4/x* | 113/159 | 0.390 | 1.43 | 0.63-3.21 | 0.217 | 0.65 | 0.34-1.28 | 0.679 | 1.19 | 0.52-2.73 |
| *HLA Bw6/6* | 98/122 | 0.584 | 1.42 | 0.41-4.95 | 0.555 | 0.74 | 0.27-2.03 | 0.755 | 0.84 | 0.27-2.57 |
| *D-R HLA-C group C1/x* | 180/237 | 0.276 | 1.53 | 0.71-3.29 | 0.330 | 0.73 | 0.38-1.37 | 0.961 | 1.02 | 0.47-2.20 |
| *D-R HLA-C group C2/2* | 31/44 | 0.493 | 0.52 | 0.08-3.29 | 0.233 | 0.36 | 0.07-1.91 | 0.321 | 0.50 | 0.13-1.94 |
| *All cases* | 211/281 | 0.324 | 1.36 | 0.73-2.52 | 0.144 | 0.69 | 0.41-1.14 | 0.703 | 1.13 | 0.59-2.15 |
| **Donor Cen-B Positive** |  |  |  |  |  |  |  |  |  |  |
| *Matched sibling donors* | 86/153 | 0.156 | 1.75 | 0.81-3.82 | 0.805 | 0.92 | 0.49-1.72 | 0.977 | 1.01 | 0.48-2.09 |
| *Matched unrelated donors* | 78/128 | 0.644 | 0.85 | 0.43-1.68 | 0.816 | 0.92 | 0.46-1.81 | 0.219 | 1.71 | 0.72-4.02 |
| *HLA-A*03/-A*11 positive* | 69/117 | 0.293 | 1.63 | 0.65-4.07 | 0.743 | 1.15 | 0.49-2.66 | 0.482 | 0.71 | 0.28-1.82 |
| *HLA-A*03/-A*11 negative* | 94/164 | 0.487 | 1.31 | 0.61-2.81 | 0.274 | 0.71 | 0.38-1.31 | 0.091 | 2.02 | 0.89-4.55 |
| *HLA Bw4/x* | 87/159 | 0.180 | 1.66 | 0.78-3.51 | 0.199 | 0.67 | 0.36-1.24 | 0.805 | 1.09 | 0.53-2.24 |
| *HLA Bw6/6* | 75/122 | 0.890 | 0.93 | 0.36-2.40 | 0.698 | 1.18 | 0.51-2.76 | 0.481 | 1.36 | 0.57-3.23 |
| *D-R HLA-C group C1/x* | 137/237 | 0.206 | 1.47 | 0.81-2.70 | 0.731 | 1.09 | 0.64-1.87 | 0.871 | 1.05 | 0.56-1.97 |
| *D-R HLA-C group C2/2* | 26/44 | 0.805 | 1.23 | 0.24-6.22 | 0.151 | 0.19 | 0.02-1.83 | 0.993 | 0.99 | 0.26-3.68 |
| *All cases* | 163/281 | 0.552 | 1.16 | 0.69-1.95 | 0.401 | 0.83 | 0.53-1.28 | 0.278 | 1.34 | 0.79-2.27 |
| **Donor Tel-B Positive** |  |  |  |  |  |  |  |  |  |  |
| *Matched sibling donors* | 71/153 | 0.773 | 0.90 | 0.44-1.83 | 0.496 | 1.27 | 0.63-2.52 | 0.966 | 0.98 | 0.47-2.02 |
| *Matched unrelated donors* | 52/128 | 0.533 | 1.28 | 0.59-2.76 | 0.147 | 0.63 | 0.33-1.17 | 0.754 | 1.13 | 0.52-2.44 |
| *HLA-A*03/-A*11 positive* | 58/117 | 0.690 | 0.83 | 0.33-2.07 | 0.520 | 1.28 | 0.61-2.69 | 0.350 | 0.67 | 0.29-1.54 |
| *HLA-A*03/-A*11 negative* | 65/164 | 0.648 | 1.18 | 0.57-2.47 | 0.460 | 0.77 | 0.39-1.52 | 0.396 | 1.38 | 0.65-2.91 |

**Table S2 (Continued.)**

| **Marker** | **N** | **Grade II-IV Acute GVHD** | | | **Chronic GVHD NST** | | | **Relapse** | | |
| --- | --- | --- | --- | --- | --- | --- | --- | --- | --- | --- |
|  |  | ***p*** | **SHR** | **95% CI** | ***p*** | **SHR** | **95% CI** | ***p*** | **SHR** | **95% CI** |
| **Donor Tel-B Positive (Continued.)** | | | | | | | | | | |
| *HLA Bw4/x* | 63/159 | 0.948 | 0.97 | 0.47-2.01 | 0.321 | 0.72 | 0.38-1.37 | 0.850 | 1.07 | 0.50-2.30 |
| *HLA Bw6/6* | 60/122 | 0.890 | 0.94 | 0.37-2.34 | 0.389 | 1.37 | 0.67-2.79 | 0.825 | 0.92 | 0.43-1.94 |
| *D-R HLA-C group C1/x* | 107/237 | 0.758 | 0.91 | 0.50-1.64 | 0.642 | 0.88 | 0.53-1.48 | 0.851 | 0.94 | 0.52-1.71 |
| *D-R HLA-C group C2/2* | 16/44 | 0.811 | 0.78 | 0.11-5.46 | 0.491 | 1.50 | 0.47-4.79 | 0.458 | 0.52 | 0.09-2.89 |
| *All cases* | 123/281 | 0.998 | 0.99 | 0.59-1.68 | 0.677 | 0.91 | 0.58-1.41 | 0.949 | 0.98 | 0.58-1.65 |
| **Donor B score ≥ 2*** | | | | | | | | | | |
| *Matched sibling donors* | 55/153 | 0.688 | 1.16 | 0.56-2.38 | 0.218 | 1.50 | 0.78-2.87 | 0.141 | 0.55 | 0.24-1.21 |
| *Matched unrelated donors* | 47/128 | 0.866 | 1.07 | 0.49-2.31 | 0.592 | 0.83 | 0.43-1.62 | 0.284 | 1.51 | 0.71-3.21 |
| *HLA-A*03/-A*11 positive* | 46/117 | 0.828 | 1.11 | 0.45-2.74 | 0.421 | 1.31 | 0.67-2.56 | 0.430 | 0.70 | 0.29-1.68 |
| *HLA-A*03/-A*11 negative* | 56/164 | 0.708 | 1.15 | 0.55-2.41 | 0.539 | 0.80 | 0.39-1.62 | 0.914 | 0.96 | 0.45-2.05 |
| *HLA Bw4/x* | 54/159 | 0.246 | 1.55 | 0.74-3.26 | 0.423 | 0.75 | 0.37-1.51 | 0.907 | 1.04 | 0.51-2.14 |
| *HLA Bw6/6* | 48/122 | 0.365 | 0.65 | 0.26-1.65 | 0.388 | 1.38 | 0.66-2.92 | 0.160 | 0.56 | 0.24-1.26 |
| *D-R HLA-C group C1/x* | 90/237 | 0.879 | 1.05 | 0.59-1.87 | 0.275 | 1.34 | 0.79-2.24 | 0.268 | 0.71 | 0.38-1.30 |
| *D-R HLA-C group C2/2* | 12/44 | 0.328 | 2.91 | 0.34-24.67 | 0.797 | 0.60 | 0.01-28.16 | 0.921 | 0.91 | 0.15-5.29 |
| *All cases* | 102/281 | 0.997 | 0.99 | 0.59-1.68 | 0.733 | 1.08 | 0.69-1.67 | 0.710 | 0.90 | 0.52-1.55 |
| **Donor B score (category 0 or 1 vs 2)^†^** | | | | | | | | | | |
| *Matched sibling donors* | 138/153 | 0.054 | 0.42 | 0.18-0.98 | 0.680 | 1.26 | 0.42-3.77 | 0.443 | 1.76 | 0.41-7.58 |
| *Matched unrelated donors* | 114/128 | 0.309 | 2.05 | 0.51-8.15 | 0.775 | 1.11 | 0.53-2.32 | 0.785 | 1.19 | 0.34-4.12 |
| *HLA-A*03/-A*11 positive* | 110/117 | 0.430 | 0.60 | 0.17-2.12 | 0.122 | 0.49 | 0.20-1.21 | 0.122 | 4.87 | 0.64-36.34 |
| *HLA-A*03/-A*11 negative* | 142/164 | 0.757 | 0.83 | 0.26-2.63 | 0.201 | 1.92 | 0.71-5.22 | 0.830 | 1.14 | 0.33-3.94 |
| *HLA Bw4/x* | 140/159 | 0.539 | 0.74 | 0.29-1.91 | 0.566 | 1.33 | 0.50-3.52 | 0.870 | 0.91 | 0.32-2.57 |
| *HLA Bw6/6* | 112/122 | 0.910 | 0.92 | 0.24-3.61 | 0.684 | 1.23 | 0.45-3.34 | - | - | - |
| *D-R HLA-C group C1/x* | 213/237 | 0.773 | 0.88 | 0.38-2.05 | 0.930 | 1.03 | 0.53-1.99 | 0.317 | 1.73 | 0.59-5.12 |
| *D-R HLA-C group C2/2* | 39/44 | 0.246 | 0.22 | 0.02-2.78 | 0.353 | 0.16 | 0.01-7.39 | - | - | - |
| *All cases* | 252/281 | 0.657 | 0.84 | 0.39-1.78 | 0.678 | 1.14 | 0.60-2.17 | 0.430 | 1.45 | 0.57-3.65 |

**Table S2 (Continued.)**

| **Marker** | **N** | **Grade II-IV Acute GVHD** | | | **Chronic GVHD NST** | | | **Relapse** | | |
| --- | --- | --- | --- | --- | --- | --- | --- | --- | --- | --- |
|  |  | ***p*** | **SHR** | **95% CI** | ***p*** | **SHR** | **95% CI** | ***p*** | **SHR** | **95% CI** |
| ***Missing Ligand Models (6, 9-12)*** | | | | | | | | | | |
| **Missing KIR2DL1 ligand** | | | | | | | | | | |
| *Matched sibling donors* | 48/111 | 0.773 | 1.15 | 0.45-2.88 | 0.075 | 0.48 | 0.21-1.07 | 0.379 | 1.39 | 0.66-2.95 |
| *Matched unrelated donors* | 49/118 | 0.369 | 0.71 | 0.33-1.49 | 0.874 | 1.06 | 0.53-2.12 | 0.987 | 1.01 | 0.45-2.20 |
| *All cases* | 97/229 | 0.990 | 0.99 | 0.57-1.72 | 0.161 | 0.69 | 0.42-1.15 | 0.552 | 1.17 | 0.68-2.02 |
| **Missing KIR2DL2/2DL3 ligand** | | | | | | | | | | |
| *Matched sibling donors* | 20/153 | 0.250 | 0.29 | 0.03-2.39 | 0.076 | 2.17 | 0.92-5.13 | 0.286 | 1.62 | 0.66-3.98 |
| *Matched unrelated donors* | 17/128 | 0.759 | 1.17 | 0.43-3.17 | 0.411 | 0.68 | 0.27-1.70 | 0.714 | 1.24 | 0.38-4.00 |
| *All cases* | 37/281 | 0.451 | 0.72 | 0.31-1.67 | 0.706 | 1.13 | 0.61-2.09 | 0.382 | 1.37 | 0.67-2.84 |
| **Missing KIR3DL1 ligand** | | | | | | | | | | |
| *Matched sibling donors* | 59/127 | 0.951 | 1.03 | 0.46-2.27 | 0.082 | 0.55 | 0.28-1.08 | 0.084 | 1.85 | 0.92-3.75 |
| *Matched unrelated donors* | 47/119 | 0.890 | 0.95 | 0.45-1.98 | 0.408 | 1.34 | 0.67-2.66 | 0.348 | 0.67 | 0.29-1.53 |
| *All cases* | 106/246 | 0.784 | 1.07 | 0.64-1.80 | 0.745 | 1.08 | 0.67-1.72 | 0.467 | 1.21 | 0.72-2.03 |
| ***KIR-Ligand Models (7, 13-15)*** |  |  |  |  |  |  |  |  |  |  |
| **Donor KIR2DL1+ Recipient HLA C1/C2** | | | | | | | | | | |
| *Matched sibling donors* | 48/111 | 0.773 | 1014 | 0.45-2.88 | 0.441 | 1.33 | 0.64-2.74 | 0.144 | 0.55 | 0.25-1.22 |
| *Matched unrelated donors* | 55/118 | 0.369 | 0.71 | 0.33-1.49 | 0.818 | 1.08 | 0.55-2.12 | 0.762 | 0.89 | 0.42-1.88 |
| *All cases* | 103/229 | 0.721 | 1.10 | 0.63-1.91 | 0.276 | 1.29 | 0.81-2.07 | 0.241 | 0.73 | 0.42-1.24 |
| **Donor KIR2DL1+ Recipient HLA C2/C2** | | | | | | | | | | |
| *Matched sibling donors* | 15/111 | 0.255 | 0.29 | 0.03-2.43 | 0.084 | 2.10 | 0.90-4.89 | 0.314 | 1.58 | 0.64-3.89 |
| *Matched unrelated donors* | 14/118 | 0.565 | 1.34 | 0.48-3.70 | 0.512 | 0.73 | 0.29-1.84 | 0.701 | 1.26 | 0.38-4.09 |
| *All cases* | 29/229 | 0.607 | 0.80 | 0.34-1.85 | 0.645 | 1.16 | 0.62-2.14 | 0.396 | 1.37 | 0.66-2.82 |
| **Donor KIR2DL2/2DL3+ Recipient HLA C1/C1** | | | | | | | | | | |
| *Matched sibling donors* | 66/153 | 0.618 | 1.25 | 0.51-3.09 | 0.067 | 0.49 | 0.23-1.05 | 0.391 | 1.38 | 0.65-2.90 |
| *Matched unrelated donors* | 52/128 | 0.375 | 0.71 | 0.34-1.48 | 0.840 | 1.07 | 0.53-2.13 | 0.942 | 1.03 | 0.47-2.25 |
| *All cases* | 118/281 | 0.887 | 1.03 | 0.60-1.77 | 0.166 | 0.71 | 0.44-1.15 | 0.551 | 1.17 | 0.68-2.02 |

**Table S2 (Continued.)**

| **Marker** | **N** | **Grade II-IV Acute GVHD** | | | **Chronic GVHD NST** | | | **Relapse** | | |
| --- | --- | --- | --- | --- | --- | --- | --- | --- | --- | --- |
|  |  | ***p*** | **SHR** | **95% CI** | ***p*** | **SHR** | **95% CI** | ***p*** | **SHR** | **95% CI** |
| **Donor KIR2DL2/2DL3+ Recipient HLA C1/C2** | | | | | | | | | | |
| *Matched sibling donors* | 67/153 | 0.777 | 1.14 | 0.45-2.88 | 0.441 | 1.33 | 0.64-2.74 | 0.144 | 0.55 | 0.25-1.22 |
| *Matched unrelated donors* | 59/128 | 0.542 | 1.25 | 0.61-2.59 | 0.764 | 1.11 | 0.57-2.15 | 0.730 | 0.87 | 0.41-1.84 |
| *All cases* | 126/281 | 0.699 | 1.11 | 0.65-1.89 | 0.263 | 1.29 | 0.82-2.05 | 0.235 | 0.72 | 0.42-1.23 |
| **Donor KIR3DL1+ Recipient HLA Bw4/4** | | | | | | | | | | |
| *Matched sibling donors* | 16/127 | 0.683 | 1.26 | 0.41-3.96 | 0.565 | 0.71 | 0.21-2.32 | 0.737 | 1.21 | 0.40-3.61 |
| *Matched unrelated donors* | 19/119 | 0.591 | 1.28 | 0.52-3.16 | 0.867 | 0.92 | 0.35-2.37 | 0.268 | 1.65 | 0.67-4.03 |
| *All cases* | 35/246 | 0.664 | 1.16 | 0.58-2.32 | 0.533 | 0.79 | 0.39-1.62 | 0.337 | 1.40 | 0.70-2.81 |
| **Donor KIR3DL1+ Recipient HLA Bw4/6** | | | | | | | | | | |
| *Matched sibling donors* | 52/127 | 0.486 | 1.49 | 0.47-4.71 | 0.111 | 1.73 | 0.88-3.41 | 0.120 | 0.56 | 0.26-1.16 |
| *Matched unrelated donors* | 53/119 | 0.715 | 1.27 | 0.34-4.64 | 0.472 | 0.79 | 0.41-1.50 | 0.905 | 1.05 | 0.48-2.27 |
| *All cases* | 105/246 | 0.572 | 0.85 | 0.49-1.47 | 0.898 | 1.03 | 0.65-1.62 | 0.172 | 0.69 | 0.41-1.17 |
| **Donor KIR2DS1+ Recipient HLA C1/C1** | | | | | | | | | | |
| *Matched sibling donors* | 21/52 | 0.880 | 0.91 | 0.25-3.24 | 0.582 | 0.68 | 0.17-2.67 | 0.818 | 1.16 | 0.32-4.14 |
| *Matched unrelated donors* | 15/43 | 0.884 | 1.08 | 0.35-3.32 | 0.707 | 0.74 | 0.15-3.57 | 0.332 | 0.46 | 0.09-2.20 |
| *All cases* | 36/95 | 0.641 | 1.21 | 0.53-2.74 | 0.263 | 0.60 | 0.24-1.46 | 0.742 | 0.84 | 0.31-2.31 |
| **Donor KIR2DS1+ Recipient HLA C1/C2** | | | | | | | | | | |
| *Matched sibling donors* | 26/52 | 0.564 | 1.47 | 0.39-5.50 | 0.972 | 0.98 | 0.34-2.75 | 0.732 | 1.26 | 0.33-4.76 |
| *Matched unrelated donors* | 22/43 | 0.755 | 0.82 | 0.24-2.79 | 0.637 | 1.42 | 0.33-5.99 | 0.829 | 1.15 | 0.32-4.13 |
| *All cases* | 48/95 | 0.749 | 0.87 | 0.38-1.98 | 0.584 | 1.23 | 0.57-2.63 | 0.679 | 1.22 | 0.47-3.12 |
| **Donor KIR2DS1+ Recipient HLA C2/C2** | | | | | | | | | | |
| *Matched sibling donors* | 5/52 | - | - | - | 0.292 | 2.37 | 0.47-11.93 | - | - | - |
| *Matched unrelated donors* | 6/43 | 0.800 | 1.23 | 0.24-6.25 | 0.811 | 0.81 | 0.14-4.42 | 0.271 | 2.96 | 0.42-20.39 |
| *All cases* | 11/95 | 0.805 | 0.83 | 0.21-3.37 | 0.312 | 1.65 | 0.62-4.35 | 0.889 | 0.89 | 0.19-4.05 |

**Table S2 (Continued.)**

| **Marker** | **N** | **Grade II-IV Acute GVHD** | | | **Chronic GVHD NST** | | | **Relapse** | | |
| --- | --- | --- | --- | --- | --- | --- | --- | --- | --- | --- |
|  |  | ***p*** | **SHR** | **95% CI** | ***p*** | **SHR** | **95% CI** | ***p*** | **SHR** | **95% CI** |
| **Donor KIR2DS2+ Recipient HLA C1/C1** | | | | | | | | | | |
| *Matched sibling donors* | 28/62 | 0.249 | 1.77 | 0.67-4.68 | 0.154 | 0.45 | 0.15-1.34 | 0.406 | 1.52 | 0.56-4.13 |
| *Matched unrelated donors* | 28/70 | 0.097 | 0.40 | 0.13-1.17 | 0.182 | 1.89 | 0.74-4.82 | 0.524 | 1.34 | 0.54-3.32 |
| *All cases* | 56/132 | 0.929 | 0.97 | 0.48-1.94 | 0.733 | 0.88 | 0.43-1.79 | 0.318 | 1.41 | 0.72-2.73 |
| **Donor KIR2DS2+ Recipient HLA C1/C2** | | | | | | | | | | |
| *Matched sibling donors* | 26/62 | - | - | - | 0.101 | 0.41 | 0.14-1.19 | 0.418 | 1.51 | 0.56-4.09 |
| *Matched unrelated donors* | 34/70 | 0.184 | 2.09 | 0.70-6.20 | 0.410 | 1.45 | 0.59-3.51 | 0.270 | 0.58 | .22-1.51 |
| *All cases* | 60/132 | 0.753 | 1.12 | 0.53-2.35 | 0.077 | 1.78 | 0.93-3.36 | 0.082 | 0.53 | 0.26-1.08 |
| **Donor KIR2DS2+ Recipient HLA C2/C2** | | | | | | | | | | |
| *Matched sibling donors* | 8/62 | - | - | - | 0.292 | 2.37 | 0.47-11.93 | - | - | - |
| *Matched unrelated donors* | 8/70 | 0.727 | 1.30 | 0.29-17.30 | - | - | - | 0.490 | 1.71 | 0.37-7.94 |
| *All cases* | 16/132 | 0.911 | 0.93 | 0.30-2.85 | 0.061 | 0.15 | 0.02-1.08 | 0.257 | 1.72 | 0.67-4.39 |
| **Donor KIR2DS3+ Recipient HLA C1/C1** | | | | | | | | | | |
| *Matched sibling donors* | 16/33 | 0.954 | 1.05 | 0.18-5.98 | 0.582 | 0.73 | 0.25-2.18 | 0.958 | 1.05 | 0.14-7.83 |
| *Matched unrelated donors* | 16/38 | 0.232 | 0.37 | 0.07-1.88 | 0.973 | 0.97 | 0.15-5.99 | 0.409 | 1.73 | 0.46-6.40 |
| *All cases* | 32/71 | 0.576 | 0.73 | 0.24-2.17 | 0.816 | 0.91 | 0.40-2.05 | 0.610 | 1.31 | 0.46-3.74 |
| **Donor KIR2DS3+ Recipient HLA C1/C2** | | | | | | | | | | |
| *Matched sibling donors* | 13/33 | 0.871 | 0.81 | 0.06-10.03 | 0.781 | 1.20 | 0.32-4.39 | 0.469 | 0.45 | 0.14-7.83 |
| *Matched unrelated donors* | 19/38 | 0.432 | 1.82 | 0.41-8.11 | 0.811 | 0.82 | 0.17-3.92 | 0.417 | 0.57 | 0.14-2.19 |
| *All cases* | 32/71 | 0.762 | 1.18 | 0.40-3.49 | 0.888 | 1.06 | 0.47-2.38 | 0.338 | 0.59 | 0.21-1.71 |
| **Donor KIR2DS3+ Recipient HLA C2/C2** | | | | | | | | | | |
| *Matched sibling donors* | 4/33 | 0.557 | 1.77 | 0.26-11.92 | 0.576 | 1.36 | 0.45-4.07 | 0.359 | 3.48 | 0.24-50.11 |
| *Matched unrelated donors* | 3/38 | 0.432 | 2.26 | 0.29-17.30 | 0.424 | 2.46 | 0.77-22.35 | 0.972 | 1.05 | 0.08-12.79 |
| *All cases* | 7/71 | 0.656 | 1.41 | 0.30-6.41 | 0.823 | 1.09 | 0.48-2.48 | 0.507 | 1.76 | 0.32-9.48 |

**Table S2 (Continued.)**

| **Marker** | **N** | **Grade II-IV Acute GVHD** | | | **Chronic GVHD NST** | | | **Relapse** | | |
| --- | --- | --- | --- | --- | --- | --- | --- | --- | --- | --- |
|  |  | ***p*** | **SHR** | **95% CI** | ***p*** | **SHR** | **95% CI** | ***p*** | **SHR** | **95% CI** |
| **Donor KIR3DS1+ Recipient HLA Bw4/4** | | | | | | | | | | |
| *Matched sibling donors* | 3/60 | - | - | - | 0.651 | 1.84 | 0.13-26.02 | - | - | - |
| *Matched unrelated donors* | 7/47 | 0.516 | 0.54 | 0.08-3.44 | 0.445 | 0.36 | 0.02-4.91 | 0.728 | 1.34 | 0.25-7.15 |
| *All cases* | 10/107 | 0.267 | 0.35 | 0.05-2.19 | 0.512 | 0.59 | 0.12-2.80 | 0.720 | 0.76 | 0.17-3.30 |
| **Donor KIR3DS1+ Recipient HLA Bw4/6** | | | | | | | | | | |
| *Matched sibling donors* | 23/60 | 0.484 | 1.49 | 0.48-4.62 | 0.150 | 1.93 | 0.78-4.76 | 0.996 | 1.00 | 0.32-3.09 |
| *Matched unrelated donors* | 22/47 | 0.750 | 1.24 | 0.31-4.87 | 0.407 | 0.54 | 0.13-2.28 | 0.855 | 0.88 | 0.21-3.52 |
| *All cases* | 45/107 | 0.586 | 1.31 | 0.48-3.54 | 0.749 | 1.12 | 0.55-2.27 | 0.912 | 1.04 | 0.46-2.36 |
| **Donor KIR3DS1+ Recipient HLA Bw6/6** | | | | | | | | | | |
| *Matched sibling donors* | 34/60 | 0.904 | 0.93 | 0.30-2.89 | 0.143 | 0.61 | 0.31-1.18 | 0.861 | 1.11 | 0.35-3.43 |
| *Matched unrelated donors* | 18/47 | 0.922 | 1.07 | 0.25-4.61 | 0.435 | 1.31 | 0.66-2.60 | 0.941 | 0.95 | 0.25-3.55 |
| *All cases* | 52/107 | 0.997 | 0.99 | 0.37-2.64 | 0.935 | 1.03 | 0.48-2.19 | 0.908 | 1.04 | 0.47-2.33 |

Abbreviations: GVHD = Graft versus host disease; NST = needing systemic therapy; SHR = sub hazard ratio (sub-distributional hazard); D = donor; R = recipient; N = number of observations (variables across various definitions described within KIR model, missing ligand model and KIR-ligand models) out of number of recipients in the corresponding cohort; “−“ denotes distributions in which statistical computation was not possible.

A multivariate competing risks regression model was used to estimate the effect of donor-recipient matching for KIR genotypes on GVHD (acute and chronic) and relapse across discovery and validation cohorts individually and in combination (all cases). The overall cohort (all cases) was also classified into donor type (matched sibling or unrelated) as well as ligand specific cohorts (for KIR models only). Ligand specific cohort stratification was based on the presence and absence of four polymorphic HLA class-I epitopes (A3/A11, Bw4, C1 and C2), which constitute ligands for KIR. A combined group of Bw4/4 and Bw4/6 was designated as Bw4/x, whereas the combined group of C1/C1 and C1/C2 recipients was designated as C1/x. Significance of association or KIR genotype matching with HCT outcomes was separately tested across donor-type and ligand-specific cohorts using the multivariate competing risks regression model. Sub-distributional hazard were described as sub-hazard ratios (SHR); p-values <0.05 were considered statistically significant (presented here in bold fonts).

*Donor B score indicates number of B-motifs in donors

†Donor B score category: 0, when b-score = 0 or 1; 1, when B-score ≥2 with cen-A/x, tel-B/x; 2, when B-score ≥2 with cen-B/B, tel-x/x

**References**

1. McQueen KL, Dorighi KM, Guethlein LA, Wong R, Sanjanwala B, Parham P. Donor-recipient combinations of group A and B KIR haplotypes and HLA class I ligand affect the outcome of HLA-matched, sibling donor hematopoietic cell transplantation. Hum Immunol. 2007;68(5):309-23.

2. Cooley S, Trachtenberg E, Bergemann TL, Saeteurn K, Klein J, Le CT, et al. Donors with group B KIR haplotypes improve relapse-free survival after unrelated hematopoietic cell transplantation for acute myelogenous leukemia. Blood. 2009;113(3):726-32.

3. Cooley S, Weisdorf DJ, Guethlein LA, Klein JP, Wang T, Le CT, et al. Donor selection for natural killer cell receptor genes leads to superior survival after unrelated transplantation for acute myelogenous leukemia. Blood. 2010;116(14):2411-9.

4. Clausen J, Wolf D, Petzer AL, Gunsilius E, Schumacher P, Kircher B, et al. Impact of natural killer cell dose and donor killer-cell immunoglobulin-like receptor (KIR) genotype on outcome following human leucocyte antigen-identical haematopoietic stem cell transplantation. Clin Exp Immunol. 2007;148(3):520-8.

5. Chen C, Busson M, Rocha V, Appert ML, Lepage V, Dulphy N, et al. Activating KIR genes are associated with CMV reactivation and survival after non-T-cell depleted HLA-identical sibling bone marrow transplantation for malignant disorders. Bone Marrow Transplant. 2006;38(6):437-44.

6. Schellekens J, Rozemuller EH, Petersen EJ, van den Tweel JG, Verdonck LF, Tilanus MG. Patients benefit from the addition of KIR repertoire data to the donor selection procedure for unrelated haematopoietic stem cell transplantation. Mol Immunol. 2008;45(4):981-9.

7. Venstrom JM, Pittari G, Gooley TA, Chewning JH, Spellman S, Haagenson M, et al. HLA-C-dependent prevention of leukemia relapse by donor activating KIR2DS1. N Engl J Med. 2012;367(9):805-16.

8. Giebel S, Nowak I, Dziaczkowska J, Czerw T, Wojnar J, Krawczyk-Kulis M, et al. Activating killer immunoglobulin-like receptor incompatibilities enhance graft-versus-host disease and affect survival after allogeneic hematopoietic stem cell transplantation. Eur J Haematol. 2009;83(4):343-56.

9. Hsu KC, Gooley T, Malkki M, Pinto-Agnello C, Dupont B, Bignon JD, et al. KIR ligands and prediction of relapse after unrelated donor hematopoietic cell transplantation for hematologic malignancy. Biol Blood Marrow Transplant. 2006;12(8):828-36.

10. Clausen J, Kircher B, Auberger J, Schumacher P, Ulmer H, Hetzenauer G, et al. The role of missing killer cell immunoglobulin-like receptor ligands in T cell replete peripheral blood stem cell transplantation from HLA-identical siblings. Biol Blood Marrow Transplant. 2010;16(2):273-80.

11. Hsu KC, Keever-Taylor CA, Wilton A, Pinto C, Heller G, Arkun K, et al. Improved outcome in HLA-identical sibling hematopoietic stem-cell transplantation for acute myelogenous leukemia predicted by KIR and HLA genotypes. Blood. 2005;105(12):4878-84.

12. Miller JS, Cooley S, Parham P, Farag SS, Verneris MR, McQueen KL, et al. Missing KIR ligands are associated with less relapse and increased graft-versus-host disease (GVHD) following unrelated donor allogeneic HCT. Blood. 2007;109(11):5058-61.

13. Cook MA, Milligan DW, Fegan CD, Darbyshire PJ, Mahendra P, Craddock CF, et al. The impact of donor KIR and patient HLA-C genotypes on outcome following HLA-identical sibling hematopoietic stem cell transplantation for myeloid leukemia. Blood. 2004;103(4):1521-6.

14. Ruggeri L, Mancusi A, Capanni M, Urbani E, Carotti A, Aloisi T, et al. Donor natural killer cell allorecognition of missing self in haploidentical hematopoietic transplantation for acute myeloid leukemia: challenging its predictive value. Blood. 2007;110(1):433-40.

15. Cooley S, Weisdorf DJ, Guethlein LA, Klein JP, Wang T, Marsh SG, et al. Donor killer cell Ig-like receptor B haplotypes, recipient HLA-C1, and HLA-C mismatch enhance the clinical benefit of unrelated transplantation for acute myelogenous leukemia. J Immunol. 2014;192(10):4592-600.
